# FedCareX: Trust-Aware Federated Transformer for Wearable IoT Seizure Prediction

**DOI:** 10.3390/s26196146

**Published:** 2026-09-28

**Authors:** Fahad Ali, Muhammad Shadab Alam Hashmi, Muhammad Ismail Mohmand, Syed Rizwan Hassan

**Affiliations:** 1 Department of Computer Science, University of Southern Punjab, Multan 59060, Pakistan; ch.fahad786@gmail.com; 2 Department of Data Science and Artificial Intelligence, Khwaja Fareed University of Engineering and Information Technology, Rahim Yar Khan 64200, Pakistan; shadab.alam@kfueit.edu.pk; 3 Department of Computer Engineering, Faculty of Engineering and Natural Sciences, Istanbul Atlas University, Istanbul 34408, Turkey; 4 Department of Computer Engineering, Gachon University, Seongnam-si 13120, Republic of Korea

**Keywords:** Internet of Things (IoT), federated learning, wearable electroencephalography, seizure prediction, trust-aware aggregation, communication efficiency, Transformer

## Abstract

Behind-the-ear electroencephalography (EEG) can now be recorded continuously outside hospital, but forecasting the preictal state is hard in a distributed deployment: raw traces cannot leave the clinical site, the wearable montage is chosen per patient, and part of the labelling comes from automated annotators rather than clinicians. Federated learning removes the need to move recordings, yet weighting each site by its sample count lets a poorly annotated site dominate the shared model. FedCareX answers this with a montage-agnostic tokenizer, a lightweight preictal Transformer encoder, an adaptive reliability aggregation rule based on annotation quality, gradient consistency and probe-set agreement, and a sparsified error-feedback codec that reduces uplink traffic. On a five-centre wearable corpus FedCareX attains 83.1±0.6% sensitivity with 0.38 false predictions per hour, a 2.8 point increase in sensitivity and a 13.6% relative reduction in false prediction rate compared with the best 2026 baseline, and on a scalp benchmark with 23 patient clients it reaches 92.8±0.4% sensitivity with 0.22 false predictions per hour. The scalp margin is significant under a paired Wilcoxon signed-rank test over 23 independent client pairs; the wearable margin rests on five centres and is reported as a hierarchical bootstrap interval of [1.1,4.4] sensitivity points rather than as a pooled significance test. Uplink volume drops by 22.1× to 0.22 MB per client per round, and edge inference spends 28.9 mJ per window against the measured 55 mJ per window budget on the gateway platform.

## 1. Introduction

Unlike other sensing nodes, which are typically deployed outdoors and for short periods, body-worn sensing nodes are deployed continuously, and there are constraints that make their deployment in healthcare challenging. The miniaturized biosignal node needs to sample hundreds of hertz for days on a low-power radio on a coin-cell budget without disrupting the patient’s daily life. Special edge-fog pipelines have been designed for this purpose, where the EEG stream is compressed losslessly and the actual samples are processed on the path, without using the wide-area link [1]. The same architectural pressure drives the broader class of resource-constrained federated IoT deployments, where the model size and update frequency that can be achieved depend on the participating device rather than the server [2]. Medical IoT federated designs have evolved into a known taxonomy of cross-silo and cross-device environments with their own convergence and privacy trade-offs [3], and systematic research into the use of reduced electrode arrays has demonstrated that only a few strategically placed electrodes can convey the vast majority of the diagnostically relevant signal [4].

These developments are directly applicable to epilepsy. The recent release of a multi-centre corpus of over 11 thousand hours of data collected from behind-the-ear EEG, electrocardiography, electromyography and movement sensors, using a single wearable device in five European monitoring centres, enables for the first time device-realistic distributed experimentation on such data [5]. The field has been moving towards end-to-end neural networks [6] from handcrafted spectral descriptors, and the most recent advances in self-supervised spectral-attention encoders [7] and temporal–spectral–spatial capsule fusion networks [8] set the record for accuracy. All of these, however, are based on a single training corpus.

This is not the case in reality. There are no institutional borders to cross for clinical EEG, so distributed training has been investigated for epilepsy, with models trained in a federated manner without sharing raw traces [9], with fog nodes as intermediaries to reduce central dependency [10], and with fully distributed topologies on low-power wearable devices [11]. More recent work suggests using module-wise weighted aggregation with personalized local updates to deal with client drift for non-identically distributed EEG [12]. Strong aggregation rules developed elsewhere, e.g., maximum correntropy aggregation, can protect the shared model from corrupted updates, but are tested on image-classification datasets rather than on physiological data [13], and in Gaussian-mixture source-free adaptation a trained predictor can be transferred to another patient without any access to the source recordings [14]. Reporting standards have also evolved, including a community-wide framework for setting data parameters, cross-validation protocol and clinically relevant metrics for algorithms of seizures from EEG [15].

The target task is defined here because detection and prediction differ. Detection recognizes a seizure already in progress and is scored against the ictal interval. Prediction, the task addressed here, must alarm while the recording still looks interictal, and is set by two clinical intervals. The seizure occurrence period (SOP) is the window in which the seizure is expected after an alarm, and an alarm is correct only if a seizure begins inside it. The seizure prediction horizon (SPH) is the interval between alarm and SOP that gives the patient or carer time to act, so no alarm may be raised inside it. A wide SOP raises sensitivity but says less about when the event will occur, and a short SPH leaves too little time to respond, so both are clinical choices rather than free hyperparameters.

This shift of preictal forecasting onto federated wearable IoT infrastructure still leaves three aspects unresolved. First, prediction is harder than detection, and the literature is rife with detection; there is no preictal label in either of the public corpora, and leakage across recording boundaries is a live possibility if onset annotations are used to infer the preictal label. Second, wearable montages are not fixed; in the multi-centre corpus the behind-the-ear electrode configuration is selected for each patient from the suspected hemisphere of onset, a patient with a lateralized suspicion providing two derivations (one same-side and one cross-head) and a patient with a generalized suspicion providing two lateral derivations, and an encoder that takes a weight column as an input would not be able to accept both. Third, and the most harmful to aggregation, the reliability of the labels is not uniform. The corpus contains a confidence value for each event, and it clearly indicates whether the annotation is from a human or an automated annotator, meaning that a rule that assigns greater weight to clients based on the number of events will amplify the influence of the client that provides the least well-annotated data.

**Figure 1:**
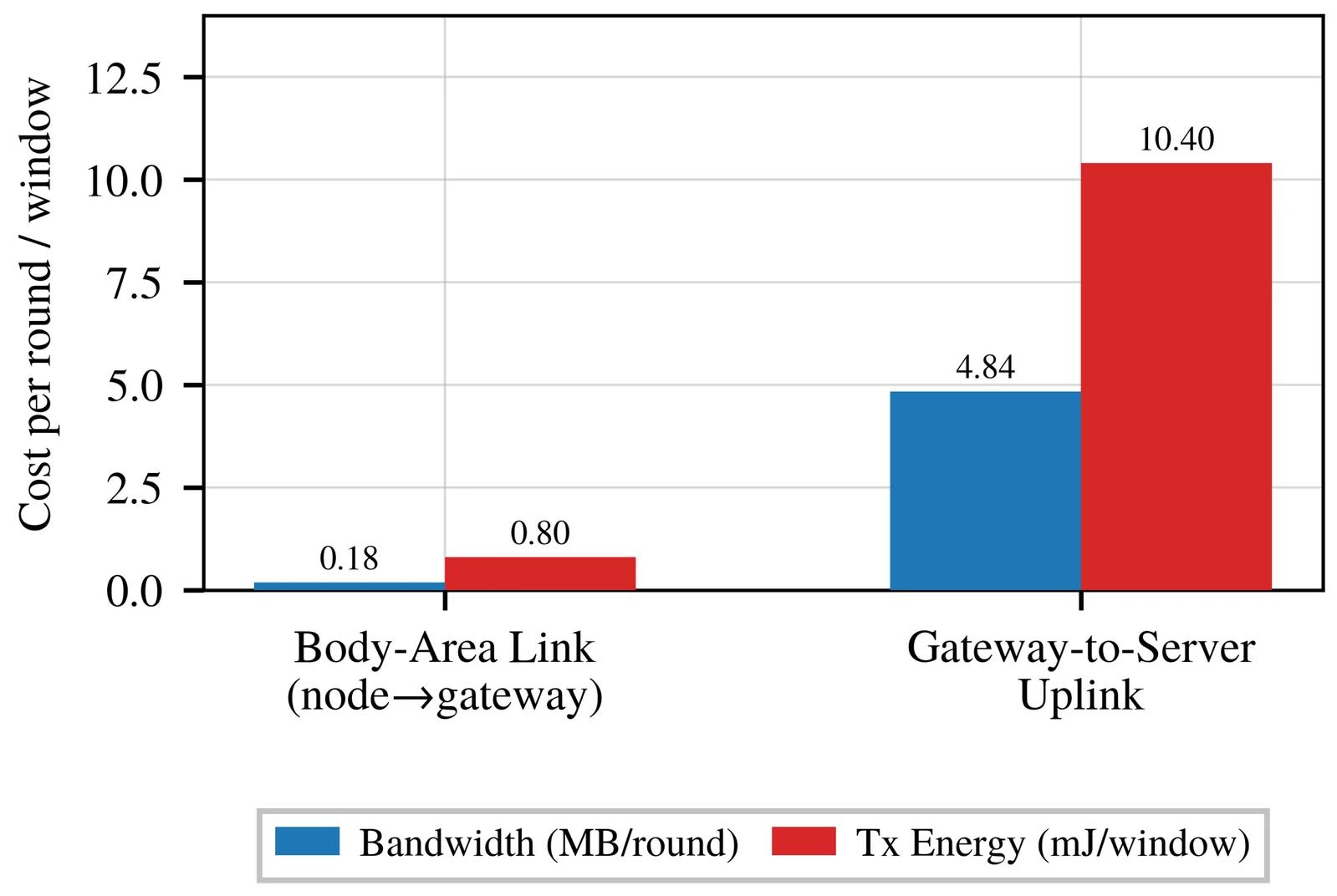
Three-tier deployment for federated wearable seizure prediction. Sensing nodes hold no model state, site gateways perform local training, and only compressed updates cross the uplink; the annotated budgets show that the gateway-to-server hop, not the body-area link, dominates the communication cost. Structure and budgets after [1,2,5,16].

**Figure 2:**
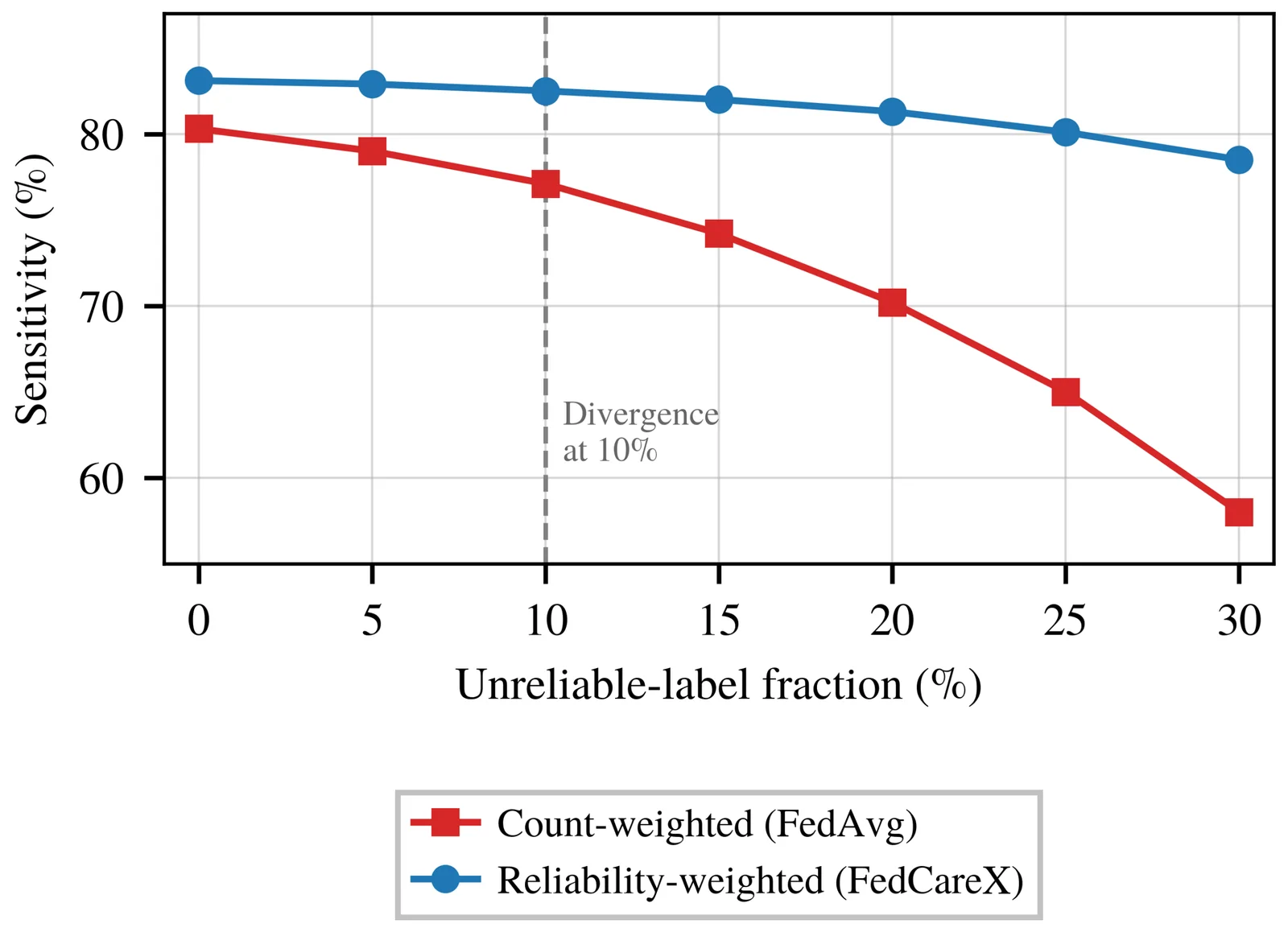
Why sample-count weighting is insufficient. As the share of weakly supervised annotations at one site grows, a count-weighted global model loses sensitivity monotonically, whereas reliability-weighted aggregation holds its operating point; the divergence begins near a 10% unreliable-label fraction. Produced by the simulated annotation-corruption protocol of Section 6, not by the main experiment.

Figure 1 makes the deployment concrete: it represents the three-tier route from the body-worn sensing node through the site gateway to the aggregation node and identifies where bandwidth and energy are actually consumed, motivating the choice to place model updates rather than raw traces on the uplink. Its tier structure and budgets follow [1,2,5,16]; it is a schematic and reports no experimental result. Figure 2 then compares the model learned when a site has poor annotation quality but is only weighted by its sample count against the model learned when the same site is down-weighted by a reliability estimate; the two trajectories diverge once the unreliable-label fraction reaches roughly one-tenth. It is a simulated corruption study on the wearable corpus: one centre has an increasing fraction of its annotations replaced by low-confidence automated labels, and the same encoder is trained twice, once with count weighting and once with the reliability weights of Section 5.1, all other settings held fixed.

The framework developed here, called FedCareX, addresses those three factors in one design. The components are not independent but reinforce each other.

- A montage-agnostic tokenizer produces a fixed-length token sequence by applying a per-channel projection to a patient-specific derivation set that is then permutation-pooled across channels, allowing for parameter-compatible updates for patients or sites where the two derivations are made from different pairs of electrodes without resorting to resampling or channel padding.
- The preictal Transformer encoder is a lightweight model built on that common representation to capture the slow spectral drift preceding the onset of a seizure, and is designed to be small enough to fit the compute and memory budget of a gateway-class edge device for a forward pass during a declared occurrence period.
- Because the encoder is shared across sites of varying quality, a reliability aggregation rule replaces weighting by the number of samples, using a composite trust score built from the confidence of the annotations, the agreement of the gradient with a robust centre, and the agreement of the locally updated model on a small server-held probe set; the score is smoothed across rounds to avoid oscillation from a single round.
- Because the estimation of trust needs multiple rounds to converge, an error-feedback codec with stochastic quantization compresses each uplink update without compromising the direction of the gradients that the trust score reads, thus achieving robustness and communication economy without sacrificing one for the other.

Both datasets were required to be publicly available, and the wearable corpus was partitioned by its recording centres. One consequence, which is not argued for here, is that no previously published federated or centralized seizure predictor reported a result on the wearable corpus, so all comparisons along that path had to be generated by reproducing the competing methods under a single protocol, as Section 6 sets out.

The rest of the paper is structured as follows. Section 2 discusses related work on Transformer-based prediction, federated physiological learning and cross-patient generalization, and states the research gap. The system model and constrained objective are formalized in Section 3. Section 4 develops the montage-agnostic tokenizer and preictal encoder and analyses their complexity. The adaptive reliability aggregation and communication codec are discussed in Section 5. Experimental evaluation is presented in Section 6 and Section 7 concludes.

## 2. Related Work

### 2.1. Transformer and Attention Models for Seizure Prediction

Attention architectures were introduced to address the short temporal receptive field of convolutional stacks in seizure prediction. A first attempt to model preictal evolution and explicitly represent label ambiguity was a hybrid vision Transformer with data uncertainty learning, which was shown to reduce false alarms from transitional segments [17]. Later, attention and convolutional families were merged: FusionXNet interleaves convolutional and Transformer blocks, learning local morphology and long-range dependency together, and enhances sensitivity on the standard splits [18]. The small size of preictal training sets is addressed by a distillation approach and by multi-branch schemes, while a multi-branch mutual-distillation Transformer transfers knowledge from branches that focus on a specific class of seizures to the other branches, boosting the accuracy on rare classes that are otherwise overwhelmed [19]. Hybrid recurrent-attention models also outperform: a model combining RNNs and Transformers shows that the recurrent component is responsible for learning slow amplitude trends that the attention component cannot smooth out [20]. The 2026 frontier is set by a self-supervised encoder that combines a multi-scale adaptive spectral block and a global-and-local temporal module to learn a joint spectral-temporal pretext representation prior to fine-tuning [7] and by TSSF-CapsNet which integrates temporal, spectral and spatial evidence using an improved global routing stage [8]. An earlier masked Transformer with adaptive frequency-time attention from the same line [21] shows that these gains come from the pretext objective rather than the classifier head. All of these encoders are trained on a single pooled corpus with one fixed montage, so none reports what happens when the corpus is split across sites that do not share an electrode layout.

Efficiency has not received as much attention as accuracy. A cross-patient detector built on a lightweight convolution Transformer showed that a small attention stack could be applied across patient populations at a small fraction of the parameter count [22]; a six-centre study of a convolution Transformer trained using a belief-matching loss is one of the few studies to assess patient-independent behaviour across genuine and well-distinguished centres [23]. Wearable systems combining ear EEG with cardiac and photoplethysmographic channels confirm that behind-the-ear acquisition carries a usable preictal signal [24], but they are trained centrally at one site. Both are centralized; what happens to this encoder when a training corpus is distributed across sites is the question addressed in Section 2.2.

### 2.2. Federated and Privacy-Preserving Learning on Physiological Data

Federated designs in physiological IoT have been evolving rapidly. A Federated Temporal Fusion Transformer for multi-horizon risk prediction in cross-silo hospitals showed that attention models can be federated and retain horizon-wise interpretability [25]. In the case of EEG, differential privacy has been incorporated into federated epilepsy recognition and the loss of accuracy from formal privacy guarantees has been measured [26]. On the systems side, the uplink rather than local compute was generally the limiting resource for an IoT federation, in particular when communication is optimized via federated multitask formulations over wireless links [16]; on the other hand, client-selection rules for various industrial deployments revealed that deciding which clients to include mattered as much as how their updates were combined [27]. This idea is extended by assigning different combination rules to different depths of the shared network via layer-wise aggregation under the control of a policy learner residing on the server [28].

These lines are mature, and the position taken here is not that they are missing. Correntropy aggregation [13] bounds the influence of an outlying update and is stronger under unbounded adversarial corruption but treats each client as a black box and cannot separate a noisy site from a merely different one. Group selection [27] and layer-wise aggregation [28] decide participation from update statistics alone, so a site whose gradients look ordinary while its labels are wrong is invisible to them, which is the failure mode discussed in Section 5.1. Quantized communication [16] is used here rather than replaced. What is added is the annotation-quality metadata that wearable corpora already publish [5] as a third evidence channel, and the requirement that compression be unbiased so the rule reading the update stays valid.

A parallel line does not federate at all; it only sends a trained model. Unlike pretrained source models that require source recordings for a given source, Gaussian-mixture modelling of the target distribution can be used to adapt a pretrained source model to a new patient even though no source recordings are available for that patient [14]; similarly, a multi-source semi-supervised variant assumes that each prior patient is an independent source, which avoids averaging out inter-patient variability among sources [29]. These are designed to protect privacy but forgo the collaborative benefit of multi-site aggregation described in the introduction, and they do not consider whether a specific source is reliable, which is the question addressed in Section 5.1.

### 2.3. Cross-Patient Generalization and Class Imbalance

The predominant error source in both centralized and distributed settings is inter-patient variability. Using source-free adaptation in the classification of subtypes further reduced the accuracy drop for unseen patients by aligning the classifier to the target distribution without target-labelled data [30], and multi-source semi-supervised schemes demonstrated that alignment was more robust when sources were kept separate than when they were combined [29].

Interictal segments greatly outnumber preictal segments, making imbalance handling essential. A small network that explicitly optimized imbalanced-learning objectives reported that re-sampling alone was insufficient and that loss re-weighting was necessary to prevent the collapse of the minority class [31]. The closest work to the montage-invariance requirement formalized in Section 3 is a channel selection approach based on multi-objective optimization that maintains predictive accuracy with a significantly smaller number of electrodes [32].

### 2.4. Research Gap

Table 1 shows that no current method provides all four capabilities. It is the foundation for the three gaps listed below, as well as the selection of comparison methods in Section 6.

**Gap 1 (prediction under federation on wearable montages).** The vast majority of federated EEG work focuses on detection [10,11], and the few federated predictors are applied to a fixed clinical scalp montage, with derivation *j* having identical physical meaning for all patients [9,12]. No patient uses a behind-the-ear derivation set with the composition and laterality studied here. The gap is filled by the montage-agnostic tokenizer of Section 4.

**Gap 2 (reliability-aware aggregation).** All federated EEG methods surveyed rely on a personalization objective [9,12,26] or are based on the number of samples. Robust aggregation is well established outside the physiological domain [13,27,28], so the gap is not the absence of such rules but the evidence they use: each scores a client from its update alone, and none exploits the annotation-confidence metadata the wearable corpus publishes for every event [5]. This gap is addressed by the adaptive reliability aggregation in Section 5.

**Gap 3 (joint robustness and communication economy).** Communication reduction in EEG federations is realized either architecturally by shrinking the model [10] or generically, that is, without reference to the robustness rule downstream [16]; the reverse is true of robustness work. The two goals have not been co-optimized for a battery-constrained wearable deployment. This gap is addressed by the error-feedback codec of Section 5.2, which is co-designed with the trust estimator so that compression does not affect the signal that it reads.

## 3. Problem Formulation and System Model

This section formalizes the federated sensing setting, the preictal labelling rule, the montage heterogeneity condition, and the constrained learning objective that the proposed framework must solve. All symbols are defined at their first point of use, and the reliability and communication quantities introduced in Section 5 follow the same client index *k* and round index *t* established here.

### 3.1. Federated Sensing Model

The deployment comprises *K* sites, K={1,…,K}. Site *k* owns a private corpus $D_{k}={\left\{\left(X_{k,i},\;y_{k,i},\;c_{k,i}\right)\right\}}_{i=1}^{n_{k}}$ with $D_{k}\cap D_{k^{\prime }}=⌀$, where $c_{k,i}$ is the annotation confidence published with the event and is the only quality metadata carried away from the site. The raw windows do not cross the site boundary. A window $X_{k,i}$ is a segment of the continuous stream extracted by a sliding operator of length *W* seconds and stride *S* seconds, so that $t_{i+1}=t_{i}+S$, with $x_{k}\left(\cdot \right)\in R^{|C_{k}|}$ the instantaneous multichannel sample.

### 3.2. Preictal Labelling

**Figure 3:**
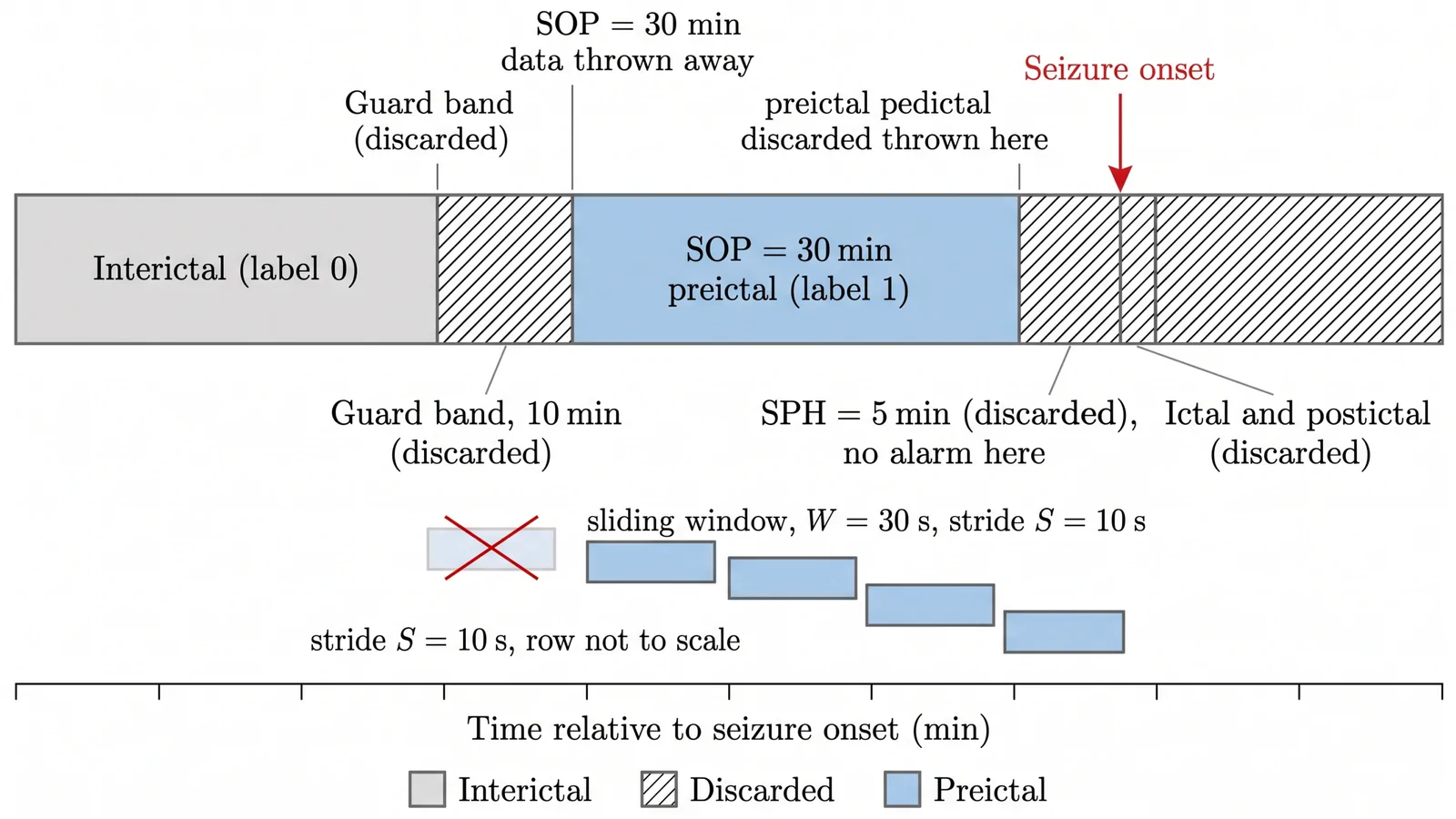
Schematic timeline of the preictal induction rule of (1), showing the interictal region, the guard band $\Delta_{b}$, the occurrence period $\Delta_{\mathrm{SOP}}$ carrying the preictal label, the horizon $\Delta_{\mathrm{SPH}}$, the onset $\tau_{o}$, and the ictal and postictal intervals. Hatched regions indicate windows discarded from training (no label assigned).

Labels for the target are induced from the onset labels of the corpus; no preictal labels are provided by either corpus. Given onset $\tau_{o}$, a seizure occurrence period $\Delta_{\mathrm{SOP}}$ and a prediction horizon $\Delta_{\mathrm{SPH}}$, the label is

(1) $$y_{k,i}=\left\{\begin{array}{cc} 1, & t_{i}\in \left[\tau_{o}-\Delta_{\mathrm{SPH}}-\Delta_{\mathrm{SOP}},\;\tau_{o}-\Delta_{\mathrm{SPH}}\right),\\ 0, & t_{i}<\tau_{o}-\Delta_{\mathrm{SPH}}-\Delta_{\mathrm{SOP}}-\Delta_{b},\\ ⌀, & \mathrm{otherwise}\;\left(\mathrm{discarded}\right), \end{array}\right.$$

where $\Delta_{b}$ removes segments next to the preictal boundary and ⌀ denotes windows to be excluded from training. If a window covers a time gap exceeding $\Delta_{\mathrm{gap}}$ or overlaps an impedance-check epoch, that window is also discarded because it contains non-physiological signal. Figure 3 shows the rule on a time axis: the guard band prevents any window from straddling the interictal-to-preictal transition, and the horizon is excluded because an alarm raised there would leave the patient no time to act.

The number of preictal windows contributed by one admissible seizure follows directly from the sliding operator and (1),

(2) $$N_{\mathrm{pre}}=\left\lfloor \frac{\Delta_{\mathrm{SOP}}-W}{S}\right\rfloor +1,$$

which reconciles the segment counts reported in Table 2 with the raw seizure counts of the two corpora. A seizure is an admissible lead seizure when four conditions hold on $I=[\tau_{o}-\Delta_{\mathrm{SPH}}-\Delta_{\mathrm{SOP}}-\Delta_{b},\;\tau_{o})$: it lies inside one recording; it contains no gap longer than $\Delta_{\mathrm{gap}}$ and no impedance epoch; no earlier annotated seizure has its onset inside it; and it does not overlap the occurrence period of a retained lead seizure. The third condition handles clustered events, since two onsets closer than $\Delta_{\mathrm{SPH}}+\Delta_{\mathrm{SOP}}+\Delta_{b}$ leave only the first retained, so no window is preictal for one seizure while lying in the postictal tail of another. This reduces 886 wearable events to 312 lead seizures and 198 scalp events to 138. The reduction is a property of preictal induction rather than of this method, since any predictor working from onset annotations must discard clustered events and incomplete lead-ins. It removes clustered seizures preferentially and retains the cleanest recordings, a mild optimism shared by every method compared here, and it transfers only where onset times are reliable; where they are not, $\Delta_{b}$ must absorb that uncertainty; the sensitivity to that choice is reported in Section 6.

**Table 2:** Segment counts after preictal induction. Preictal counts follow from (2). The table is reproducible from the raw corpora using a single 1:8 lead-seizure to interictal draw ratio, and no further filtering is applied. Source information for every input quantity is given in Table 3.

Property	SeizeIT2	CHB-MIT
Acquisition	Behind-the-ear wearable	Clinical scalp
Sampling rate (Hz)	250	256
Derivations per patient	2 (composition varies)	23 common
Recordings retained	all	683 of 686
Federated clients	5 (centres)	23 (patients)
Patients	125	23
Recorded hours	11,640	≈1000
Annotated seizures	886	198
Admissible lead seizures	312	138
Preictal windows per seizure	178	178
Preictal segments	55,536	24,564
Interictal segments	444,288	196,512
Preictal share (%)	11.11	11.11
Annotation confidence	Published, 0–1	Absent, set to 1
Active trust terms	$q_{k}$, $g_{k}$, $v_{k}$	$g_{k}$, $v_{k}$
$\Delta_{\mathrm{SOP}}$/$\Delta_{\mathrm{SPH}}$ (min)	30/5	30/5
Guard $\Delta_{b}$/gap $\Delta_{\mathrm{gap}}$ (min)	10/1	10/1
Window *W*/stride *S* (s)	30/10	30/10
Evaluation protocol	Leave-one-centre-out	Leave-one-subject-out

**Table 3:** Provenance of the dataset quantities used in this study. The third column differs from the second only where a filtering rule of this section is applied; such differences are not corrections to the sources. All values were read from the released corpora and reconstructed from the raw recordings.

Corpus	Quantity	As Released by the Source	As Used in This Study
SeizeIT2	Release, licence	ds005873 v1.1.0, BIDS 1.8.0, CC0; DOI 10.18112/openneuro.ds005873.v1.1.0	Identical; ethics UZ Leuven S63631
	Cohort	125 patients, 5 European units, 1 paediatric	5 federated clients; sex and age not used as covariates
	bte-EEG derivations	2 per patient, composition layout-dependent	2, treated as an unordered set (Section 4.2)
	Focal seizures	886 annotated	312 admissible lead seizures after applying (2)
	Annotation confidence	Range 0–1, human or automated annotator	$c_{k,i}$, drives (15)
	Impedance epochs	Marked artefactual by the corpus	Discarded (Algorithm 1, line 3)
CHB-MIT	Recordings, cases	686 EDF files, 24 cases, 23 unique subjects	683 files after excluding chb12_27–29 for a divergent montage; 23 clients
	Annotated seizures	198 annotated	138 admissible lead seizures after applying (2)
	Annotation schema	Seizure start and end times only	$c_{k,i}=1$; (15) inactive on this track
Both	File integrity	Published checksum manifest	Verified before ingestion, then signals re-derived from the raw EDF/BIDS streams

**Algorithm 1** Local Preictal Training at Site *k* **Require:**Global parameters $\theta^{\left(t\right)}$; local corpus $D_{k}$; epochs *E*; proximal coefficient μ; gap tolerance $\Delta_{\mathrm{gap}}$ **Ensure:**Local update $\Delta_{k}^{\left(t\right)}$; quality score $q_{k}^{\left(t\right)}$  1:$\theta_{k}\leftarrow \theta^{\left(t\right)}$  2:$D_{k}\leftarrow$ sliding windows labelled by (1)  3:discard windows crossing a gap $>\Delta_{\mathrm{gap}}$ or an impedance epoch  4:retain only lead seizures admissible under (2)  5:**for** e=1 **to** *E* **do**  6:   **for all** minibatch $B\subset D_{k}$ **do**  7:     $H^{\left(0\right)}\leftarrow$ tokens by (8)–(10)  8:     $H^{\left(L\right)}\leftarrow$ encoder stack with (11)  9:     $\hat{y}\leftarrow$ prediction by (12)10:    $L_{k}\leftarrow$ objective by (13) with proximal term11:     update $\theta_{k}$ by AdamW12:   **end for**13:**end for**14:$q_{k}^{\left(t\right)}\leftarrow$ confidence statistic by (15)15:$\Delta_{k}^{\left(t\right)}\leftarrow \theta_{k}-\theta^{\left(t\right)}$                                 ▷ (14)16:**return** $\Delta_{k}^{\left(t\right)},\;q_{k}^{\left(t\right)}$

### 3.3. Montage Heterogeneity

The layouts of wearable behind-the-ear devices can be selected for each patient depending on the suspected onset hemisphere; thus, $C_{k}$ is not constant across sites, nor is it constant within a site. The two layouts have the same cardinality but are physically different: a generalized suspicion gives a left and a right lateral derivation, while a lateralized suspicion gives a same-side derivation and a cross-head derivation. Writing $C={⋃}_{k\in K}C_{k}$ for the union of layouts,

(3) $$\exists \;k,k^{\prime }\in K:\;C_{k}\neq C_{k^{\prime }},\;\left|C_{k}\right|\leq C_{\max},\;\;\forall k,$$

where the relation is on sets and not on cardinalities, so (3) is satisfied when two clients have different numbers of derivations and when they have the same number but different pairs of electrodes. The wearable corpus used here is of the second type. This is the technical reason why a shared input layer cannot bind a weight column to a derivation index: it would be trained on a cross-head signal at one patient versus a lateral signal at another. Condition (3) is what the tokenizer of Section 4.2 must satisfy.

### 3.4. Learning Objective

With $R_{k}\left(\theta \right)$ the local empirical risk of site *k* under the per-window loss *ℓ* specified in (13), conventional federation combines the per-site risks into the count-weighted sum

(4) $$\min_{\theta }\;\;R\left(\theta \right)=\sum_{k\in K}\frac{n_{k}}{\sum_{j}n_{j}}\;R_{k}\left(\theta \right).$$

The weight in (4) depends only on cardinality, which is precisely the deficiency identified in Figure 2: a site that provides many weakly supervised windows outweighs a site that provides fewer but more reliable ones.

### 3.5. Communication, Energy and Latency

With |θ| parameters, a retained fraction ρ and *b* bits per retained coordinate, each surviving coordinate must carry both its value and its position, so the per-client uplink volume in round *t*, and the edge energy per processed window, are

(5) $$B^{\left(t\right)}=\rho \;\left|\theta \right|\;\left(b+\lceil \log_{2}|\theta |\rceil \right)/8\;\mathrm{bytes},$$

(6) $$E_{k}=\underset{\mathrm{compute}}{\underset{︸}{\kappa \;F(\theta )}}+\underset{\mathrm{transmit}}{\underset{︸}{P_{\mathrm{tx}}\;\frac{8\;B^{\left(t\right)}}{R_{k}\;\left|B_{k}\right|}}},$$

where F(θ) is the forward-pass operation count, κ the per-operation energy of the gateway, $P_{\mathrm{tx}}$ the radio power, $R_{k}$ the uplink rate in bits per second and $|B_{k}|$ the number of windows the client processes per round. The uncompressed reference is recovered by setting ρ=1, b=32 and dropping the index term, which gives 4|θ| bytes. The two components of (6) are reported separately in Section 6 because they respond to different design levers. End-to-end inference latency $\Lambda_{k}$ sums acquisition, preprocessing and the forward pass and excludes any server round trip, because inference is local; it is therefore a distinct quantity from the per-round wall-clock time $\Lambda_{k}^{\mathrm{rd}}=\Lambda_{k}^{\mathrm{train}}+8B^{\left(t\right)}/R_{k}$, which covers local training plus uplink occupancy and is the quantity tabulated under round latency.

The design problem is therefore the constrained programme

(7) $$\begin{array}{cc} \min_{\theta ,\;\{\omega_{k}\}}\; & \sum_{k\in K}\omega_{k}\;R_{k}\left(\theta \right)\\ s.t.\; & \sum_{k}\omega_{k}=1,\;\;\omega_{k}\geq 0,\;\;B^{\left(t\right)}\leq B_{\max},\;\;E_{k}\leq E_{\max},\;\;\Lambda_{k}\leq \Lambda_{\max}. \end{array}$$

The weights $\omega_{k}$ in (7) are treated as decision variables rather than as fixed constants, which is the structural difference from (4); their determination is the subject of Section 5, and the budgets it carries are what the codec of Section 5.2 must respect. Reported gateway budgets in constrained federated IoT deployments are of the same order, which confirms that these are realistic rather than nominal limits [2,16].

## 4. Proposed Methodology

### 4.1. Architecture Overview

**Figure 4:**
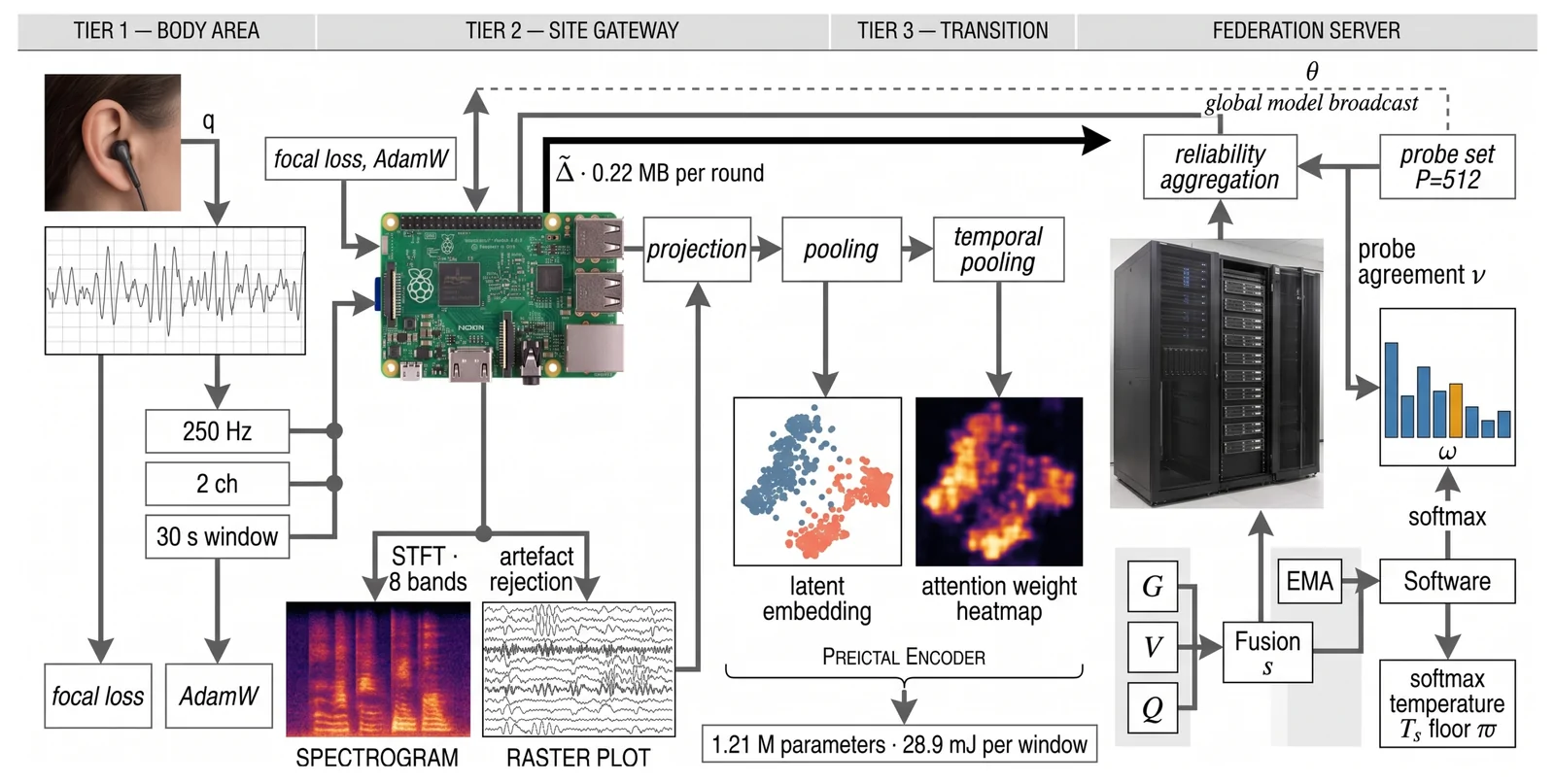
FedCareX architecture. The montage-agnostic tokenizer absorbs per-patient channel variation before any shared weight is touched, the preictal encoder is identical at every site, and the server combines updates through reliability weights derived from three independent evidence sources rather than from sample counts. Solid arrows denote data flow; dashed arrows denote weight/control flow.

**Figure 5:**
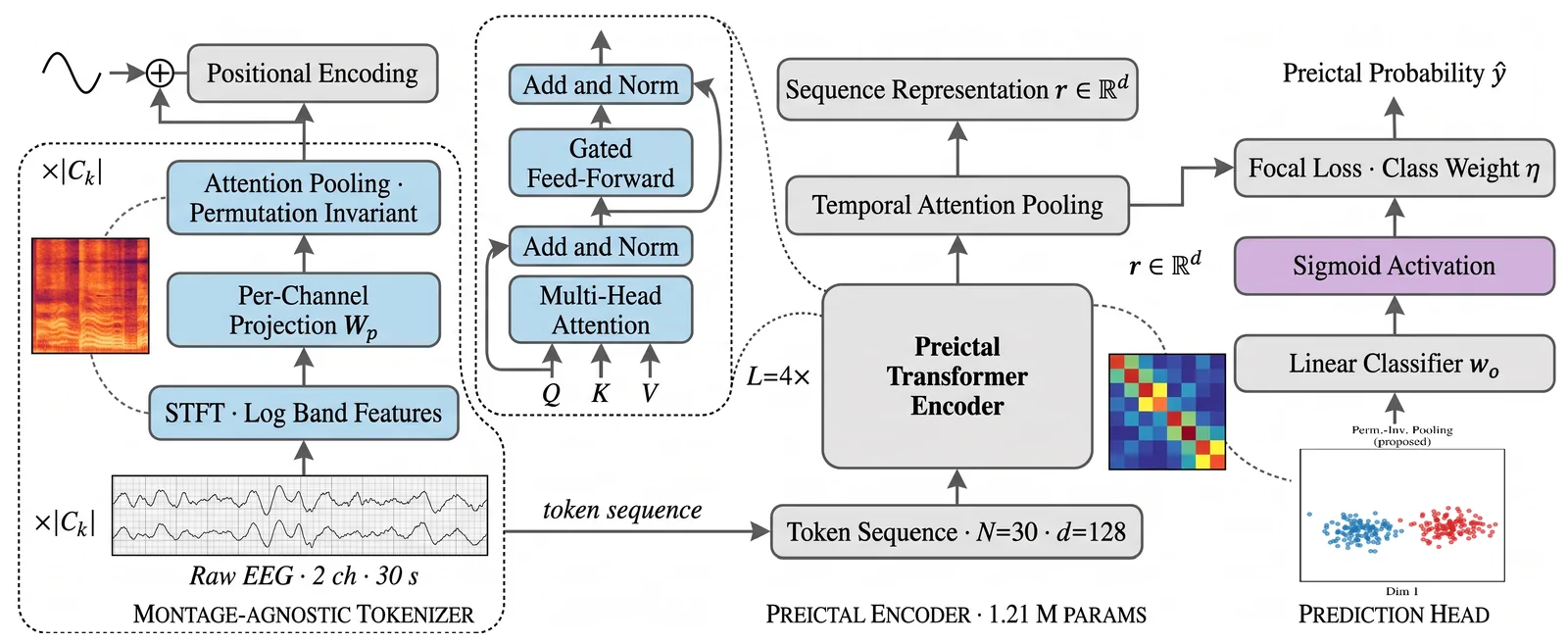
Internal architecture of FedCareX showing the montage-agnostic tokenizer, preictal Transformer encoder with expanded layer detail, and sigmoid prediction head with focal loss. Arrows indicate the forward-pass data flow through each sub-module.

Figure 4 shows the complete framework and the direction of every data and control flow: it identifies which quantities remain inside a site, which cross the uplink, and where the trust estimator draws its three inputs from, so that the reader can verify that no raw window and no per-window label leave the client. Figure 5 expands the internal structure of the tokenizer, the encoder stack and the prediction head, which is the view needed to follow Algorithms 1 and 2.

### 4.2. Montage-Agnostic Tokenizer

Each retained window is first mapped to a spectrogram $S_{c}$ by a short-time Fourier transform with a Hann window of length *M* and hop $H_{s}$. Bins are compressed logarithmically and aggregated into *F* physiologically motivated bands,

(8) $$z_{c}\left[j,\tau \right]=\log(\epsilon_{0}+\sum_{f\in F_{j}}S_{c}\left[f,\tau \right]),\;j=1,\ldots ,F,$$

where $\epsilon_{0}$ prevents singularities at silent bins. The band edges cover the range in which preictal spectral drift is observed, and (8) is the only place where domain priors enter the representation.

Channel variation is absorbed by a single projection $W_{p}\in R^{F\times d}$ that is shared by all channels of all sites, followed by attention pooling with a learned query $a\in R^{d}$,

(9) $$u_{c,\tau }=W_{p}^{⊤}\;z_{c}\left[:,\tau \right]+b_{p}\in R^{d},$$

(10) $$\alpha_{c,\tau }=\frac{\exp\;\left(a^{⊤}\tanh\left(u_{c,\tau }\right)\right)}{\sum_{c^{\prime }\in C_{k}}\exp\;\left(a^{⊤}\tanh\left(u_{c^{\prime },\tau }\right)\right)},\;{\tilde{u}}_{\tau }=\sum_{c\in C_{k}}\alpha_{c,\tau }\;u_{c,\tau }.$$

The shape of $W_{p}$ does not depend on $|C_{k}|$; this is why (9) is applied on a per-channel basis rather than to a concatenation. The operation in (10) is permutation-invariant over channels and defined for any $|C_{k}|\geq 1$, so it returns the same representation for any two derivations of a patient, whether same-side and cross-head or left and right lateral. This is exactly what condition (3) demands: the pooling discards the ordering and identity of derivations, which then need not be shared across sites. Adding standard sinusoidal positional encodings to ${\tilde{u}}_{\tau }$ restores ordering across time frames and gives the token sequence $H^{\left(0\right)}$ of fixed length N=T.

This invariance has a cost. The pooled representation cannot express which side of the head a change came from, so laterality is unavailable to the encoder. It is not part of the label here, since the target is a binary preictal decision, and the per-frame weights $\alpha_{c,\tau }$ still let the derivation carrying the stronger drift dominate, so what is lost is its identity rather than its contribution. The limitation is that the encoder cannot support a lateralization output, and would discard useful structure on a dense clinical montage where derivation *j* has a fixed anatomical meaning across patients.

**Figure 6:**
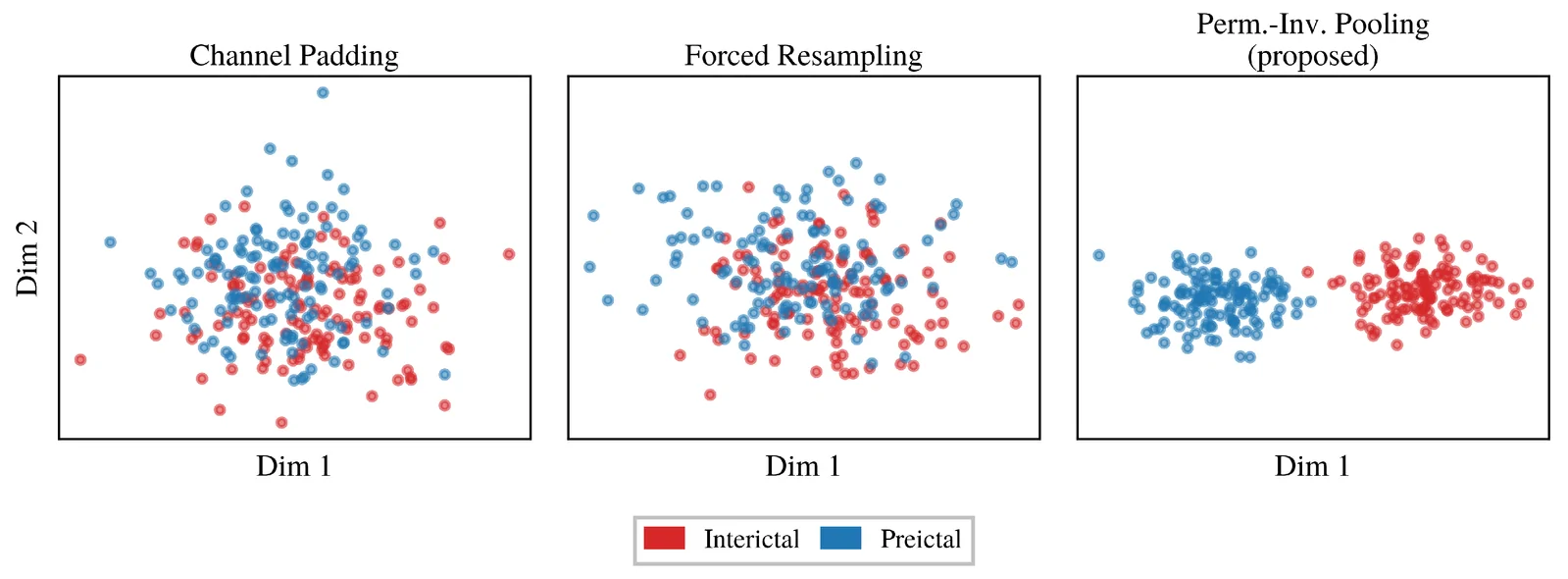
Justification for montage-agnostic pooling. Channel padding and forced resampling both collapse preictal and interictal embeddings once the electrode layout varies within a client, whereas permutation-invariant pooling preserves the margin.

Figure 6 empirically justifies this component by comparing the separability of preictal and interictal embeddings under channel padding, resampling to a common montage, and pooling as in (10), and shows that pooling is the only one of these that achieves separability under a layout that varies within a cohort.

### 4.3. Preictal Transformer Encoder

The encoder is a pre-norm Transformer of depth *L* with *H* heads. Queries, keys and values are the usual linear projections of $H^{\left(\ell -1\right)}$, and the *H* head outputs are concatenated and mixed by $W_{\ell }^{O}$ to form MHA(·), with attention logits scaled by $\sqrt{d/H}$ because a gateway-sized model has little room for gradient instability. The only departure from the standard block is a gated feed-forward stage, composed residually with the attention output,

(11) $$\mathrm{FFN}\left(h\right)=W_{2}\;\left(\sigma \left(W_{g}h\right)⊙\left(W_{1}h\right)\right)+b_{2},$$

in which σ(·) denotes the logistic function throughout this paper, so that ${\hat{H}}^{\left(\ell \right)}=H^{\left(\ell -1\right)}+\mathrm{MHA}\left(\mathrm{LN}\left(H^{\left(\ell -1\right)}\right)\right)$ and $H^{\left(\ell \right)}={\hat{H}}^{\left(\ell \right)}+\mathrm{FFN}\left(\mathrm{LN}\left({\hat{H}}^{\left(\ell \right)}\right)\right)$. Sequence reduction uses the same attention-pooling form as (10), now over time with query $a_{t}$,

(12) $$r=\sum_{\tau =1}^{N}\beta_{\tau }\;h_{\tau }^{\left(L\right)},\;\beta_{\tau }=\frac{\exp\;\left(a_{t}^{⊤}\tanh\left(h_{\tau }^{\left(L\right)}\right)\right)}{\sum_{\tau^{\prime }}\exp\;\left(a_{t}^{⊤}\tanh\left(h_{\tau^{\prime }}^{\left(L\right)}\right)\right)},$$

and the preictal probability is $\hat{y}=\sigma \left(w_{o}^{⊤}r+b_{o}\right)$. Reusing the pooling form of (10) in (12) keeps the parameter budget low, a cost that multi-branch and capsule predictors incur through additional routing stages [8,19].

The depth and width are not claimed optimal in the unconstrained sense. They are the smallest values at which sensitivity saturates under the per-window budget $E_{\max}$ of (7); Section 6 reports configurations above that budget, so the cost of the constraint is visible rather than assumed.

### 4.4. Local Objective

The interictal windows far outnumber the preictal windows by about an order of magnitude, and hence a class-weighted focal cross-entropy is used, where the annotation confidence enters as a per-sample factor,

(13) $$\ell \left(\hat{y},y\right)=-\;c_{k,i}\left[\eta \;y{\left(1-\hat{y}\right)}^{\gamma }\log\hat{y}+\left(1-y\right)\;{\hat{y}}^{\gamma }\log\left(1-\hat{y}\right)\right],$$

in terms of the class-balance coefficient η and the focusing parameter γ. The form is asymmetric by construction: η appears only in the minority term. By placing $c_{k,i}$ into (13), a site can locally discount its own uncertain annotations. This is a different use of the confidence signal from the aggregation weight of (15): the former reweights samples within a fixed client and cannot change the client’s participation in the global update, while the latter reweights whole clients. Section 6 reports the ablation that separates the two effects. To reduce the risk of client drift a proximal term $\frac{\mu }{2}{∥\theta -\theta^{\left(t\right)}∥}_{2}^{2}$ is added to the local risk, and the resulting objective is minimized by *E* epochs of AdamW at the settings listed in Section 6, after which the transmitted quantity is the displacement

(14) $$\Delta_{k}^{\left(t\right)}=\theta_{k}^{\left(t\right)}-\theta^{\left(t\right)}.$$

Only (14) leaves the site, and it is the object that the codec of Section 5.2 compresses and the trust estimator of Section 5.1 inspects. The coefficients μ, γ and η are swept in Section 6.

### 4.5. Complexity

Per window the tokenizer costs $O(|C_{k}|\;M\log M\;T)$ for the transform and $O(|C_{k}|\;F\;d\;T)$ for (9), and the pooling of (10) is never dominant. The encoder stack costs $O(L\left(N^{2}d+Nd^{2}\right))$ in operations and $O(L\left(Nd+d^{2}\right)+Fd)$ in memory. With *N* only a few tens the quadratic term is small against $Nd^{2}$, so the encoder stays inside $E_{\max}$ when substituted into (6), and the memory term is independent of $|C_{k}|$ by construction of (9). Algorithm 1 therefore runs in $O(E|D_{k}|F\left(\theta \right))$ time, and Algorithm 2 adds O(K|θ|) for aggregation plus a negligible probe term. Unlike channel-selection designs that obtain patient robustness from a fixed subset of a fixed montage [32], the invariance here is structural and applies to all shared weights, which is why sites with incompatible layouts can all feed into one parameter vector.
**Algorithm 2** FedCareX Communication Round **Require:**$\theta^{\left(t\right)}$; client set K; probe set P; ratio ρ; bit width *b*; smoothing λ **Ensure:**$\theta^{\left(t+1\right)}$  1:**for all** k∈K **in parallel do**  2:   $(\Delta_{k}^{\left(t\right)},q_{k}^{\left(t\right)})\leftarrow$ Algorithm 1  3:   ${\tilde{\Delta }}_{k}^{\left(t\right)}\leftarrow$ Compress $\left(\Delta_{k}^{\left(t\right)},\rho ,b\right)$                ▷ Algorithm 3  4:   transmit $\left({\tilde{\Delta }}_{k}^{\left(t\right)},q_{k}^{\left(t\right)}\right)$  5:**end for**  6:$\Delta^{★}\leftarrow$ robust centre by (16)  7:**for all** k∈K **do**  8:   $g_{k}^{\left(t\right)}\leftarrow$ (17);    $v_{k}^{\left(t\right)}\leftarrow$ (18)  9:   $s_{k}^{\left(t\right)}\leftarrow$ (19);    ${\bar{s}}_{k}^{\left(t\right)}\leftarrow$ (20)10:**end for**11:$\{\omega_{k}^{\left(t\right)}\}\leftarrow$ weights by (21)12:$\theta^{\left(t+1\right)}\leftarrow \theta^{\left(t\right)}+\sum_{k}\omega_{k}^{\left(t\right)}{\tilde{\Delta }}_{k}^{\left(t\right)}$                    ▷ (22)13:**return** $\theta^{\left(t+1\right)}$


**Algorithm 3** Trust-Coupled Compression and Weighting
 **Require:**$\Delta_{k}^{\left(t\right)}$; accumulator $e_{k}^{\left(t-1\right)}$; ratio ρ; bits *b*; scores $q_{k}^{\left(t\right)}$; probe set P; thresholds $g_{\min},v_{\min}$ **Ensure:**${\tilde{\Delta }}_{k}^{\left(t\right)}$, $\omega_{k}^{\left(t\right)}$  1:**function** Compress$\left(\Delta_{k}^{\left(t\right)},\rho ,b\right)$  2:     $p_{k}^{\left(t\right)}\leftarrow \Delta_{k}^{\left(t\right)}+e_{k}^{\left(t-1\right)}$; $m_{k}^{\left(t\right)}\leftarrow$ top-ρ mask  3:     ${\tilde{\Delta }}_{k}^{\left(t\right)}\leftarrow Q_{b}\left(m_{k}^{\left(t\right)}⊙p_{k}^{\left(t\right)}\right)$; $e_{k}^{\left(t\right)}\leftarrow p_{k}^{\left(t\right)}-{\tilde{\Delta }}_{k}^{\left(t\right)}$                ▷ (23), (24)  4:     **return** ${\tilde{\Delta }}_{k}^{\left(t\right)}$  5:
**end function**
  6:**at server:** $\Delta^{★}\leftarrow$ Weiszfeld iterations for (16)  7:$g_{k}^{\left(t\right)}\leftarrow$ (17);    $v_{k}^{\left(t\right)}\leftarrow$ (18)  8:**if** $g_{k}^{\left(t\right)}<g_{\min}$ **and** $v_{k}^{\left(t\right)}<v_{\min}$ **then**  9:     flag client *k* for reduced participation in the next round10:
**end if**
11:$s_{k}^{\left(t\right)}\leftarrow$ (19);    ${\bar{s}}_{k}^{\left(t\right)}\leftarrow$ (20);    $\omega_{k}^{\left(t\right)}\leftarrow$ (21)12:**return** ${\tilde{\Delta }}_{k}^{\left(t\right)},\;\omega_{k}^{\left(t\right)}$



## 5. Adaptive Reliability Aggregation and Communication Codec

### 5.1. Trust Estimation

The weights $\omega_{k}$ left undetermined in (7) are estimated from three sources, each of which can flag anomaly on its own; a site must therefore be anomalous in two or more ways before it is heavily discounted. The first is the annotation metadata already published with the corpus, summarized at site *k* by a single scalar,

(15) $$q_{k}^{\left(t\right)}=\frac{1}{n_{k}}\sum_{i=1}^{n_{k}}c_{k,i}\;\cdot \;\left(1-\frac{\left|\{i:\;c_{k,i}<c_{\mathrm{th}}\}\right|}{n_{k}}\right),$$

where the second factor punishes the tail of low-confidence events, not just the mean; transmitting it reveals two aggregate statistics but no window-level information. The second is the agreement of the update direction with the population: a geometric-median centre is computed over decompressed updates by a few Weiszfeld iterations, and consistency is the rectified cosine similarity to that centre,

(16) $$\Delta^{★}=\arg\min_{z}\sum_{k\in K}∥z-{\tilde{\Delta }}_{k}^{\left(t\right)}{∥}_{2},$$

(17) $$g_{k}^{\left(t\right)}=\max\;\left(0,\;\frac{\langle {\tilde{\Delta }}_{k}^{\left(t\right)},\;\Delta^{★}\rangle }{∥{\tilde{\Delta }}_{k}^{\left(t\right)}{∥}_{2}\;{∥\Delta^{★}∥}_{2}}\right).$$

Because the geometric median is not swayed by extreme updates, the reference in (16) cannot be pulled by a single outlier, as a count-weighted average would be. The third source is behavioural rather than geometric: the server holds a small probe set P, and the probe score is the exponentiated negative balanced probe loss of the locally updated model,

(18) $$v_{k}^{\left(t\right)}=\exp\;\left(-\frac{1}{\left|P\right|}\sum_{(X,y)\in P}\ell \left(f_{\theta^{\left(t\right)}+{\tilde{\Delta }}_{k}^{\left(t\right)}}\left(X\right),\;y\right)\right).$$

Because (18) evaluates a model rather than inspects data, it detects a site whose update is geometrically ordinary but semantically wrong, which (17) alone would not detect.

The probe set is specified here because it is an input to the method. Its windows come from a public reference corpus that is not the corpus under evaluation in that run, so no probe window is seen by a client and no probe subject appears in a test partition. They are induced by (1) and drawn class-balanced, so (18) cannot be minimized by a constant predictor, and the set is fixed at 512 windows before the first round and never updated. This makes an assumption explicit: the server must hold a small labelled set. That is weaker than centralizing training data, but real, and a deployment that cannot meet it falls back on two axes at the cost reported in Section 6.

The three scores are multiplied so that a low value on any single axis mutes the client, smoothed across rounds to dampen oscillation from one bad minibatch, and mapped to weights by a tempered normalization at temperature $T_{s}$ that retains a floor ϖ on every participant,

(19) $$s_{k}^{\left(t\right)}={\left(q_{k}^{\left(t\right)}\right)}^{a_{1}}{\left(g_{k}^{\left(t\right)}\right)}^{a_{2}}{\left(v_{k}^{\left(t\right)}\right)}^{a_{3}},\;\sum_{j}a_{j}=1,$$

(20) $${\bar{s}}_{k}^{\left(t\right)}=\lambda \;{\bar{s}}_{k}^{\left(t-1\right)}+\left(1-\lambda \right)\;s_{k}^{\left(t\right)},\;{\bar{s}}_{k}^{\left(0\right)}=1,$$

(21) $$\omega_{k}^{\left(t\right)}=\left(1-K\varpi \right)\;\frac{\exp\;\left({\bar{s}}_{k}^{\left(t\right)}/T_{s}\right)}{\sum_{j\in K}\exp\;\left({\bar{s}}_{j}^{\left(t\right)}/T_{s}\right)}+\varpi ,$$

(22) $$\theta^{\left(t+1\right)}=\theta^{\left(t\right)}+\sum_{k\in K}\omega_{k}^{\left(t\right)}\;{\tilde{\Delta }}_{k}^{\left(t\right)},$$

so that (22) is the concrete version of the objective (7) with the weights instantiated. Not every corpus publishes annotation confidence; when it does not, all $c_{k,i}$ are unity, (15) returns one for every client, and (19) reduces to a two-term product with the remaining exponents renormalized. This is the case for the scalp track and is the reason its margin is expected to be smaller. With ϖ=0.02 the uniform component absorbs aggregation mass Kϖ equal to 0.10 on the wearable track and 0.46 on the scalp track, so the trust-driven softmax has a smaller absolute effect there. The floor also implements the soft exclusion applied in Algorithm 3, for the same reason that heterogeneous IoT federations prefer graded selection to a binary rule [27,28]: a site can be temporarily inconsistent as it takes on a new group of patients.

Two properties are stated because the exponents are fixed by hand. They are not tuned on the evaluation fold: the adopted mixture is selected once on the scalp track and transferred unchanged, so the design sweep in Section 6 reports the shape of the plateau rather than the search behind the operating point; every mixture inside that plateau lands within 0.6 sensitivity points of the adopted one, while any single axis alone loses 2.2 to 2.9. No convergence claim is made. What holds is bounded: by (21) every weight lies in [ϖ,1−(K−1)ϖ] and the weights sum to one, so (22) is a convex combination that cannot amplify any update beyond the largest received; (23) is unbiased; and (20) is a contraction with factor λ. Under the assumptions standard for federated averaging the scheme therefore behaves as averaging with fixed bounded weights, and a rate accounting for the dependence introduced by (20) is left open.

**Figure 7:**
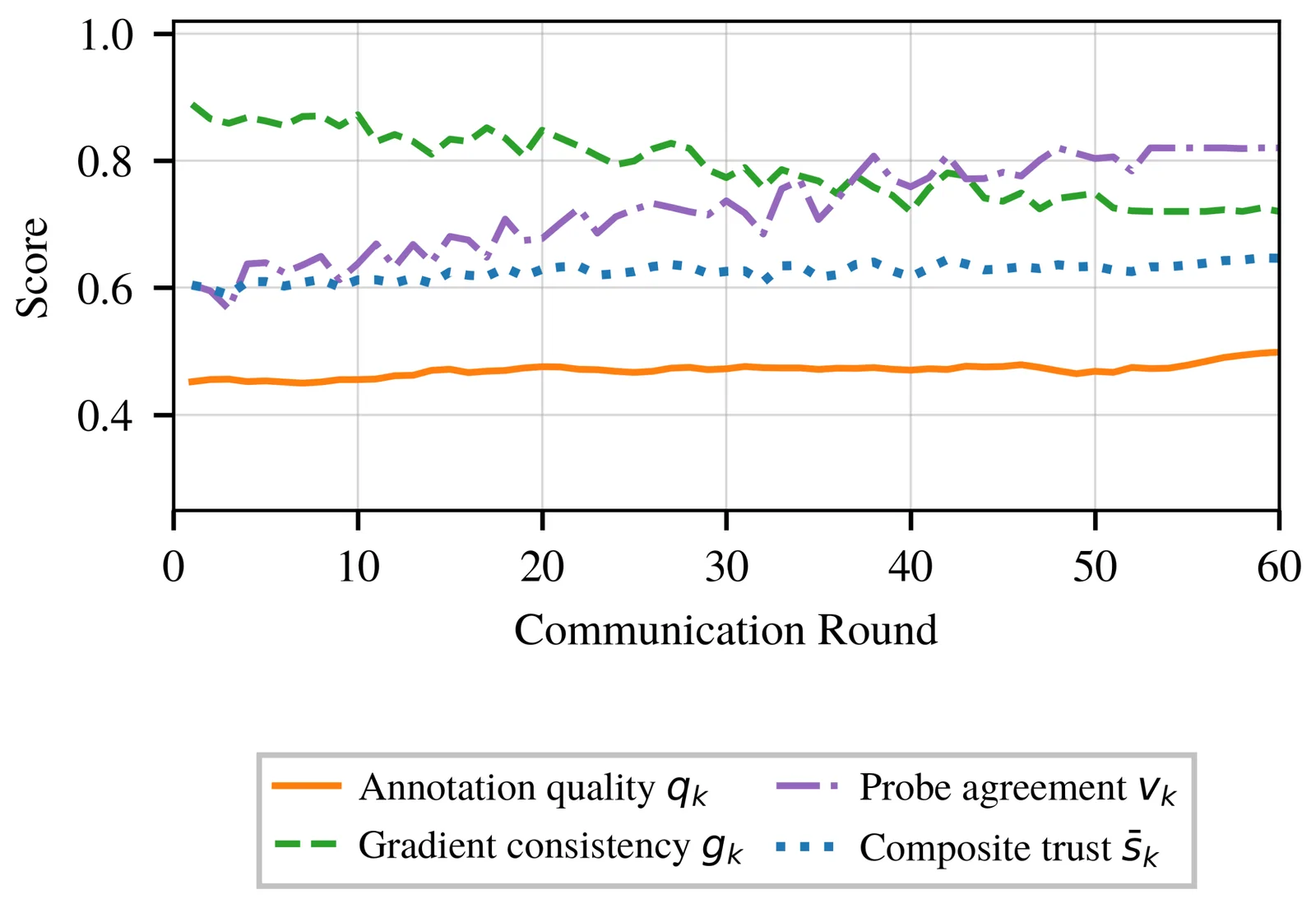
Justification for multiplicative trust fusion. Gradient consistency remains near nominal for a site with degraded annotations, so only a product form in which any single depressed factor suppresses the weight isolates that site. Scores recorded under the simulated annotation-corruption protocol of Section 6.

The multiplicative form is justified in Figure 7, which shows the three component scores for a site whose annotation quality is degraded and demonstrates that the geometric term alone remains near nominal, meaning that an additive combination would fail to isolate the site. It comes from the corruption protocol of Section 6, not the main comparison.

### 5.2. Communication Codec

Compression is applied prior to transmission but must retain the direction information read in (17). The residual accumulator $p_{k}^{\left(t\right)}=\Delta_{k}^{\left(t\right)}+e_{k}^{\left(t-1\right)}$ is first updated from $e_{k}^{\left(0\right)}=0$, and a magnitude mask $m_{k}^{\left(t\right)}$ retains the coordinates whose magnitude reaches the (1−ρ) quantile $\pi_{\rho }\left(p_{k}^{\left(t\right)}\right)$, that is, the largest ρ fraction. Retained coordinates are quantized with the standard uniform dithered quantizer,

(23) $$Q_{b}\left(u\right)=\mathrm{sgn}\left(u\right)\;{∥p_{k}^{\left(t\right)}∥}_{\infty }\;\frac{1}{2^{b}-1}\left\lfloor \frac{\left(2^{b}-1)|u\right|}{∥p_{k}^{\left(t\right)}{∥}_{\infty }}+\xi \right\rfloor ,$$

with ξ∼U[0,1), so that $E[Q_{b}\left(u\right)]=u$ and the reconstructed update is unbiased, the same property used in quantized distributed optimization for bandwidth-limited federations [16]. The transmitted object and the carry-over accumulator are

(24) $${\tilde{\Delta }}_{k}^{\left(t\right)}=Q_{b}\left(m_{k}^{\left(t\right)}⊙p_{k}^{\left(t\right)}\right),\;e_{k}^{\left(t\right)}=p_{k}^{\left(t\right)}-{\tilde{\Delta }}_{k}^{\left(t\right)}.$$

**Figure 8:**
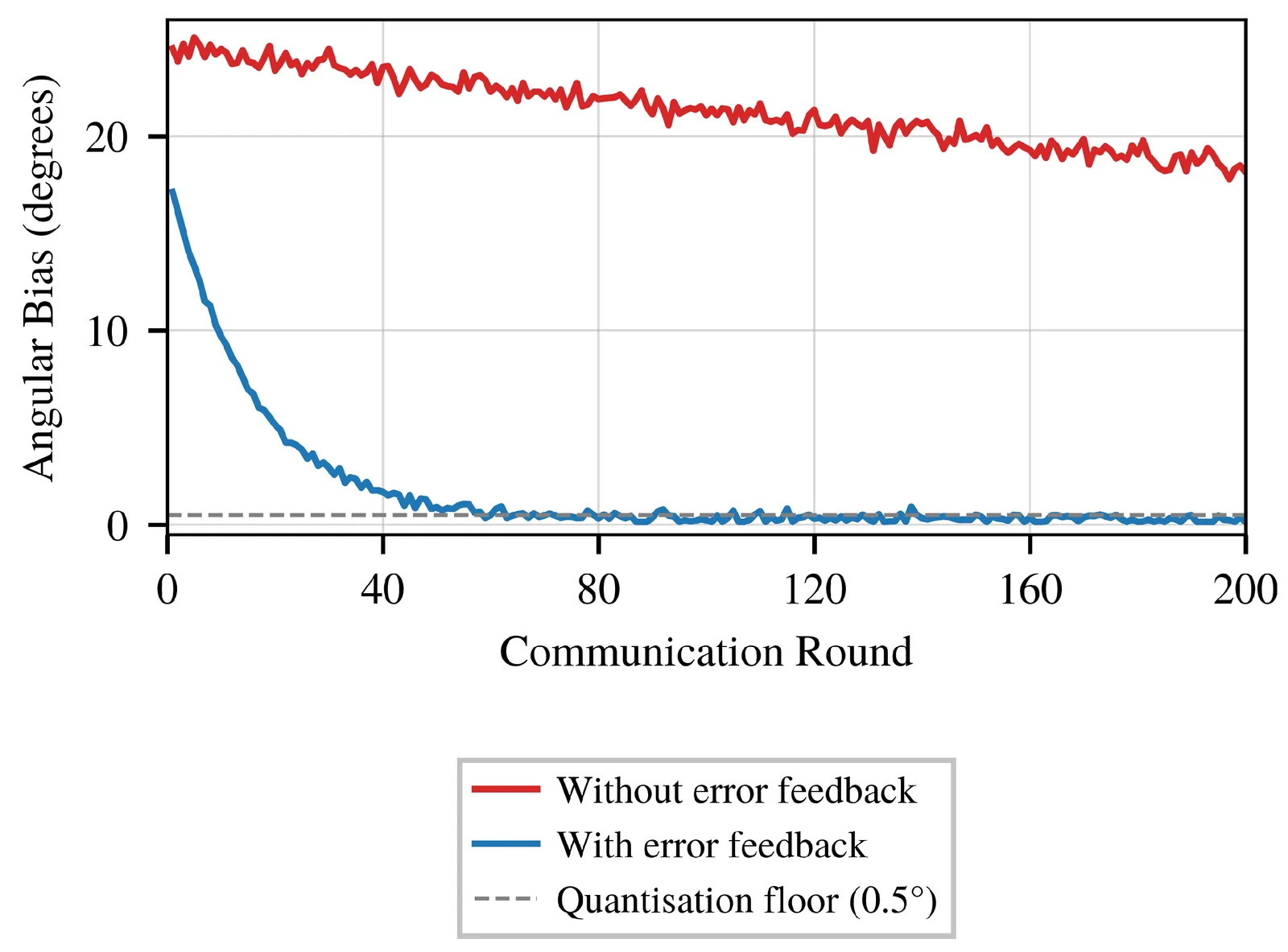
Justification for error feedback. Without the residual accumulator the aggregated update retains a persistent angular bias relative to the uncompressed direction; with it the bias decays to the quantization floor within roughly forty rounds.

Unbiasedness of (23) preserves the cosine estimated in (17) during compression, so a site is not penalized by codec error; (24) guarantees that suppressed coordinates are never lost but eventually transmitted. Substituting the retained fraction into (5) gives the per-round volume reported in Section 6. Figure 8 justifies the error-feedback path by showing that the angular bias otherwise present cannot be eliminated by additional rounds.

## 6. Results and Discussion

### 6.1. Datasets

Two publicly available corpora are used, presented separately because their acquisition hardware and montages are not directly compatible.

**SeizeIT2** https://openneuro.org/datasets/ds005873 (accessed on 15 June 2025) (v1.1.0, BIDS 1.8.0, HED-SCORE 1.1.0, CC0) provides 11 640 h of recordings from 125 focal-epilepsy patients (51 women) collected in five European monitoring units in Belgium, Germany, Sweden and Portugal (with two centres in one country), at 250 Hz on a 6.3 g body-worn EEG device. The five centres serve directly as the five federated clients, giving a partition drawn from clinical practice rather than synthetic splitting; only one centre enrols paediatric cases, which is the sole source of the cohort asymmetry examined in this section. The 886 annotated seizures have a mean duration of 58 s and a maximum of 16 min, so the longest seizure is still shorter than the 30 min occurrence period. The 10 min guard $\Delta_{b}$ drops any window straddling the preictal-interictal boundary; ictal and postictal samples fall into the discarded clause of (1). Two annotation fields drive the method: a per-event confidence in [0,1] that also records whether the annotator is human or automated, which becomes $c_{k,i}$ and the sole basis of (15), and an impd event type marking impedance-check epochs discarded by Algorithm 1.

**CHB-MIT** https://physionet.org/content/chbmit/1.0.0/ (accessed on 15 June 2025) contains 686 scalp EEG recordings at 256 Hz across 24 cases, of which 141 hold at least one of the 198 annotated seizures. The 24 cases correspond to 23 unique subjects (chb01 and chb21 are merged as the same subject recorded eighteen months apart); 683 recordings are retained after excluding chb12_27–chb12_29 for a divergent montage, and the 23 derivations common to all retained recordings are used as input. The corpus reports only seizure onset and offset times, so $c_{k,i}=1$ throughout, (15) is inactive, and the trust score (19) operates on its two remaining axes with exponents renormalized to 0.5:0.5; CHB-MIT therefore provides a partial test of the reliability rule.

Segment counts in Table 2 follow from (2) with $\Delta_{\mathrm{SOP}}=1800$ s, W=30 s and S=10 s, giving 178 preictal windows per admissible seizure; SeizeIT2 supplies 312 lead seizures and CHB-MIT 138 by the same criterion. A single 8:1 interictal-to-preictal draw is applied on both tracks, fixing the preictal share at 11.11% and staying well within the candidate windows admitted by the recording durations (4.19 M wearable, ≈0.32 M scalp). This imbalance motivates the class-weighting in (13).

### 6.2. Evaluation Metrics

Following community reporting practice [15], four clinically meaningful metrics are reported. Sensitivity is the proportion of seizures with at least one alarm inside the occurrence period, and the false prediction rate is the number of alarms raised outside occurrence periods divided by the total interictal hours. Event-level rather than window-level accounting is used because a window-level score would be skewed by repeated alarms within a single event. The area under the receiver operating characteristic and the F1 score are also reported for comparability with window-level literature.

Each configuration is trained five times with different random seeds. The across-seed standard deviation (SD) is reported in the text and tables; it quantifies run-to-run variation and is a different and much smaller quantity than the across-centre spread shown in the per-client analysis later in this section, which is reported separately and is not an error bar. A two-sided paired Wilcoxon signed-rank test over per-client scores is used to assess significance. On the scalp track the 23 patient clients provide 23 truly independent pairs. On the wearable track there are only five centres and the values from different seeds within a centre are not independent, so the 25 pairs are not independent and the pooled test overstates the effective sample size. To bound that effect, the wearable margin is additionally assessed by a hierarchical bootstrap that resamples centres with replacement and then seeds within centre, carried out with more than $10^{4}$ replicates. The bootstrap places the sensitivity margin over [7] at 2.8 points with a 95% interval of [1.1,4.4]; the margin therefore excludes zero under the conservative treatment, but the width of that interval, not the pooled *p*-value, is the honest measure that five centres can support.

### 6.3. Implementation

Table 4 lists the training environment and the edge platforms on which the energy and latency numbers are measured. The current-sense front end sampled at 100 kHz, and power was integrated over the entire window-to-decision period and averaged over 500 windows to yield the energy consumed per window, with the measured idle draw of the same board subtracted.

The 55 mJ figure is a measurement on the two gateway-class platforms of Table 4 and is not a universal wearable budget. It is a budget for the site gateway, the tier that performs local training in Figure 1, and not for the sensing node, which runs no model at all and whose envelope is smaller by orders of magnitude. Comparable constrained federated IoT deployments report budgets of the same order [2,16], but a different system-on-chip or radio would shift both the total and the ratio between the two terms of (6), which is why they are tabulated separately in the communication and energy analysis later in this section.

### 6.4. Comparison Methods

Eight published methods are reproduced under the same partition, preictal induction rule, loss and evaluation protocol as this study, with 200 rounds and five seeds, so that decentralized and fog-assisted topologies are neither advantaged nor penalized. The set covers two federated preictal predictors (Spiking-GCNN with ANFIS [9], FedMWAD [12]), two federated detectors re-targeted to (1) without altering the encoder (fog-assisted Fed-ESD [10], decentralized Res1DCNN [11]), two rules imported from adjacent domains (the source-free GSFDA [14] and the maximum correntropy aggregation MCA [13], the latter re-hosted on our tokenizer to isolate the aggregation rule), and two 2026 centralized architectures federated with count weighting so that only their encoders are compared (TSSF-CapsNet [8] and the self-supervised spectral-temporal transformer of Huang et al. [7], whose pretext stage runs on each client’s unlabelled interictal data before federated fine-tuning). All rows reproduce published architectures and aggregation rules rather than restate published scores.

No baseline is held to a parameter budget: each is federated at the size published by its authors, and each transmits at its own compression setting, with the resulting volumes reported in the communication and energy analysis later in this section. What the protocol fixes is the induction rule, the partition, the loss and the number of rounds, because a comparison in which those differ measures the protocol rather than the method. The remaining asymmetry is that the proposed encoder was designed under the gateway energy constraint while the baselines were not, and the design-choice analysis at the end of this section reports what it achieves when that constraint is lifted.

Table 5 lists the original task, paradigm, corpora and headline numbers for each baseline. No prior method reports on the wearable corpus used here, and the source works report incompatible metric pairs: only [14] publishes the event-level pair used here, while [7,9] report window-level accuracy and specificity and [10,11] report specificity and geometric mean. Table 6 therefore reproduces every number under a common protocol rather than copying figures from any source paper.

### 6.5. Overall Performance

**Figure 9:**
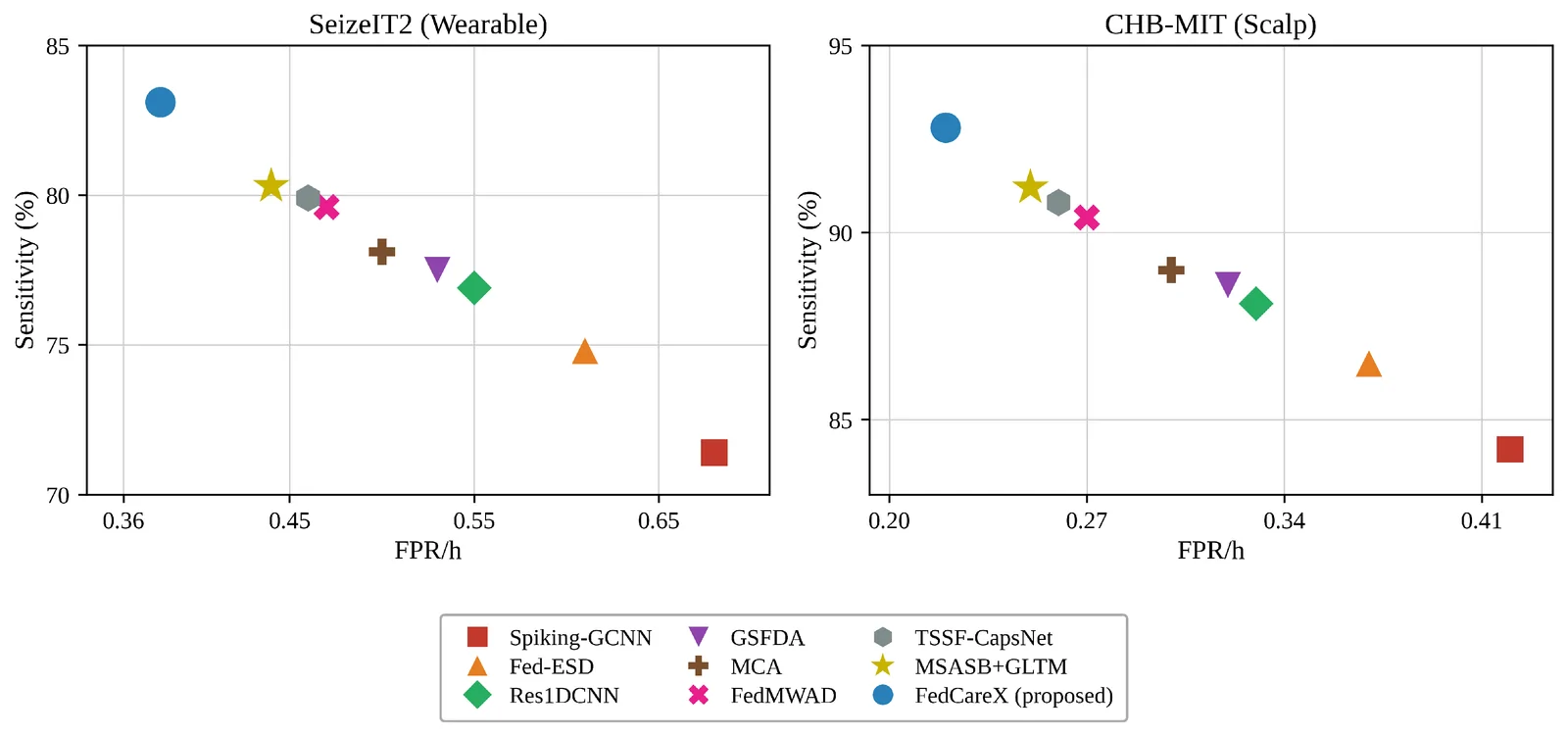
Operating points on both tracks. FedCareX reaches 83.1% sensitivity at 0.38 false predictions per hour on the wearable corpus and 92.8% at 0.22 on the scalp corpus, dominating every baseline in both coordinates simultaneously. Baselines: Spiking-GCNN with ANFIS [9], Fed-ESD [10], Res1DCNN [11], GSFDA [14], MCA [13], FedMWAD [12], TSSF-CapsNet [8], MSASB with GLTM [7].

Table 6 shows that the two 2026 architectural baselines [7,8] attain the highest scores among prior methods on both tracks and are still behind FedCareX by more than the gap between the two of them. Figure 9 plots the sensitivity and false-prediction-rate columns of that table for easy comparison of operating points.

Across five seeds FedCareX attains 83.1±0.6% sensitivity on the wearable track against 80.3±0.7% for the strongest baseline [7], and 92.8±0.4% against 91.2±0.5% on the scalp track. All quoted deviations are across seeds at a fixed data partition and not across clients. The two-sided paired Wilcoxon signed-rank test on the 23 independent client pairs of the scalp track gives p=0.002; on the wearable track the same test on the 25 centre-seed pseudo-replicate pairs gives p=0.004, but the pairs are not independent and the more honest measure is the hierarchical bootstrap interval [1.1,4.4] sensitivity points introduced in the previous subsection.

The behind-the-ear track is also harder than the scalp track, and this is instructive rather than accidental: with only two derivations versus 23 on the scalp montage, there is far less spatial redundancy, so any technique that depends on spatial diversity loses more. The proposed margin is larger on the harder track (2.8 sensitivity points versus 1.6), which has two reinforcing causes. The wearable corpus is the one with heterogeneous annotation quality, exactly the setting Figure 2 predicts should matter, and it is the only one of the two that releases the annotation-confidence metadata on which (15) depends. On the scalp track the trust score operates on two axes instead of three, so a smaller margin is expected and is not a sign of a weaker mechanism. That scalp margin is nonetheless obtained with the quality term inactive, which shows the two remaining axes carry part of the effect and that the rule does not depend on metadata only one corpus publishes. The margin is expected to be narrow because both 2026 baselines are strong encoders tested under the same preictal induction rule; a comparable margin under matched conditions is a more informative comparison than a larger margin under unequal conditions.

### 6.6. Per-Client Behaviour

**Figure 10:**
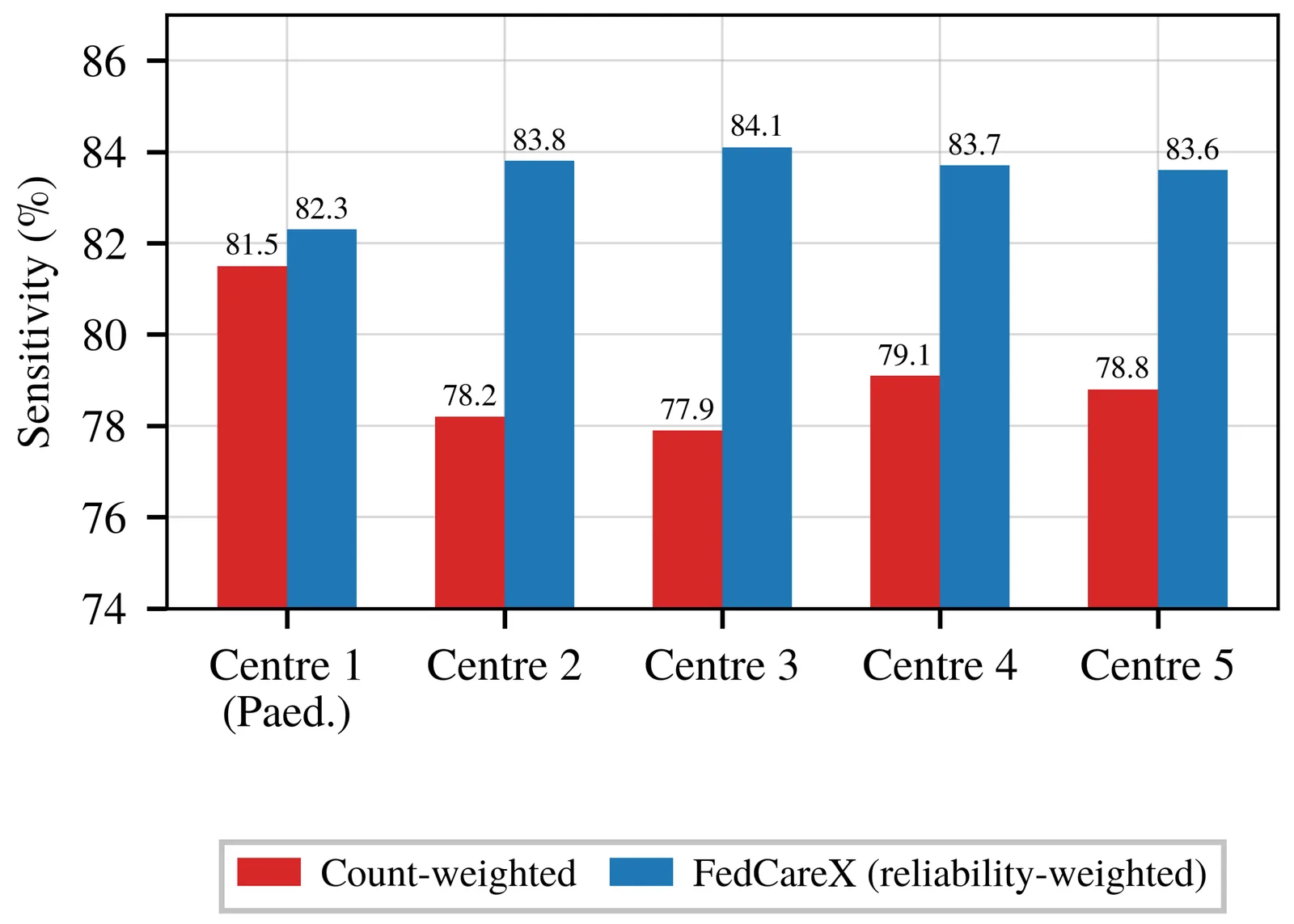
Per-centre sensitivity on the wearable corpus. The two smallest centres gain most, confirming that count weighting rather than data scarcity was limiting them.

**Figure 11:**
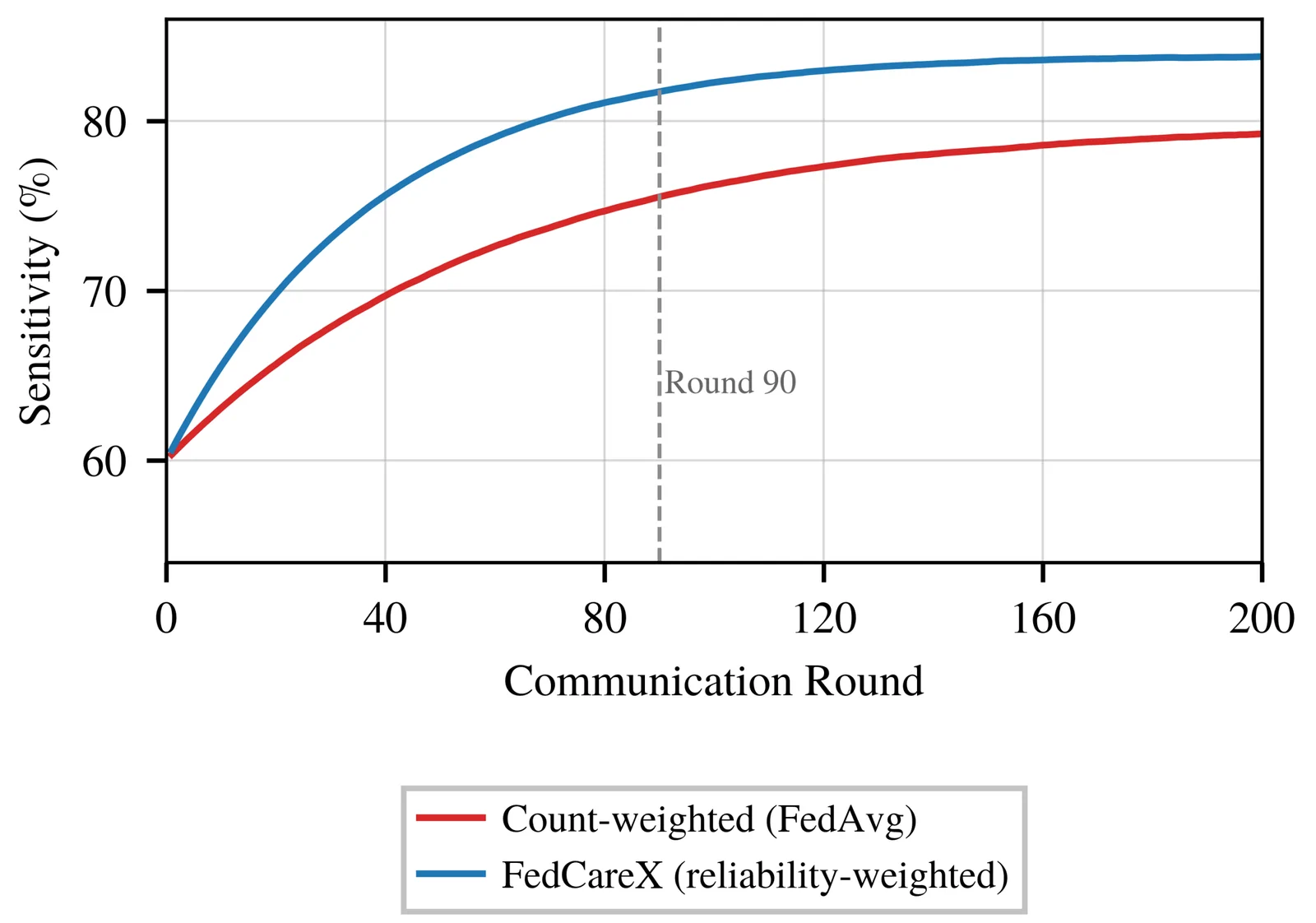
Convergence under the two weighting rules. Reliability weighting attains the count-weighted terminal accuracy at round 90 and continues improving, so the benefit is not merely a better endpoint.

Figure 10 breaks down the wearable outcome and shows that no single site dominates the improvement. The single centre that enrols paediatric patients also accounts for the largest proportion of windows and benefits least in absolute terms; the two smallest centres benefit most, which is exactly the outcome that count weighting was suppressing. The across-centre spread in the figure is large compared with the across-seed deviation reported above, and the two are not synonymous: the former captures residual heterogeneity between sites after aggregation, while the latter captures run-to-run variation at a fixed partition. Figure 11 shows the corresponding training dynamics, with reliability weighting reaching the count-weighted final accuracy in about 90 rounds instead of 200.

### 6.7. Robustness to Unreliable Clients

**Figure 12:**
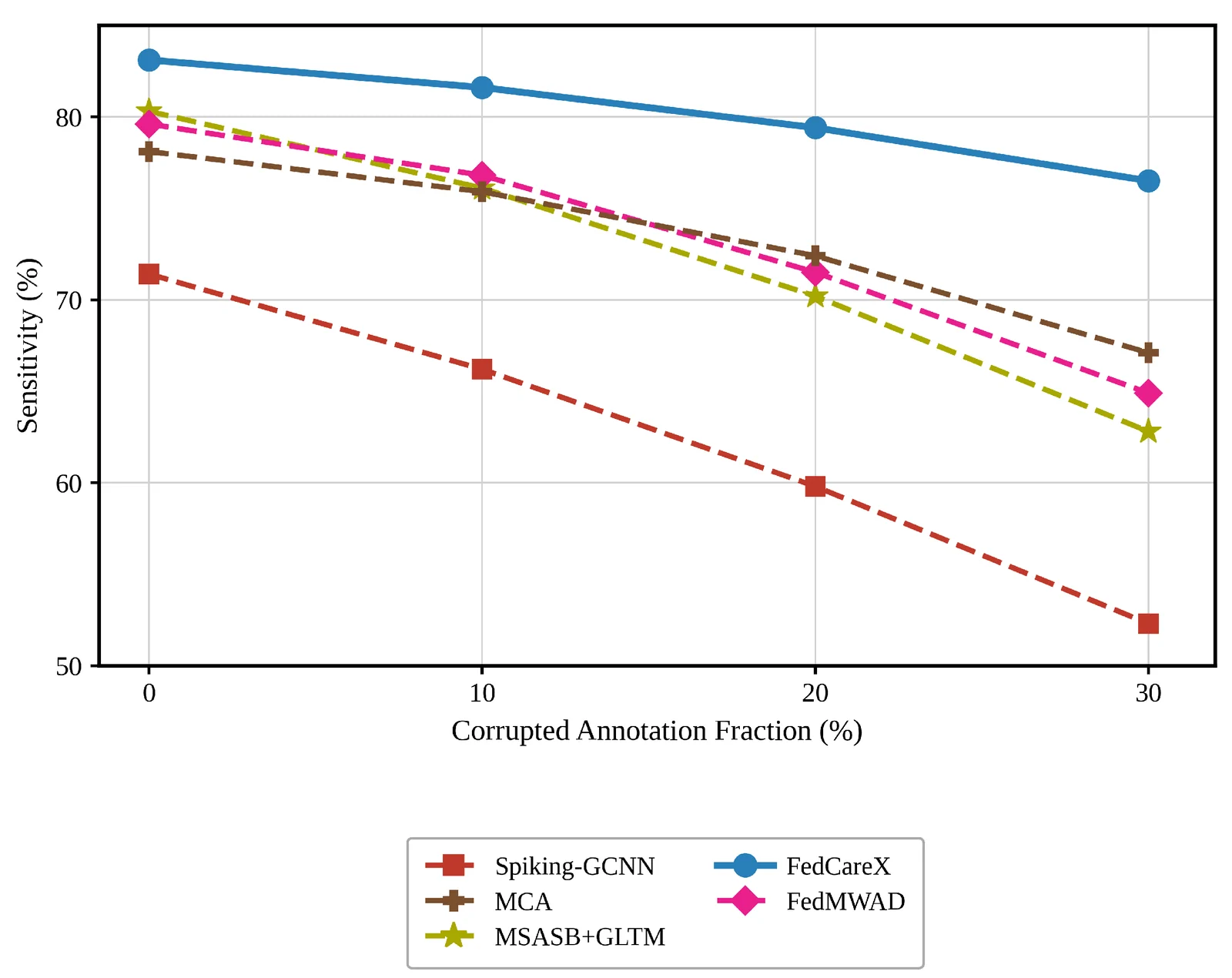
Degradation under annotation corruption. At a 30% corrupted fraction FedCareX retains 76.5% sensitivity, a 6.6-point loss across the sweep, against losses of 11.0 to 19.1 points for the reproduced baselines: Spiking-GCNN with ANFIS [9], MSASB with GLTM [7], FedMWAD [12], MCA [13].

Table 7 reports the degradation curve: the proportion of bad annotations is raised at one wearable centre, and Figure 12 plots it. This experiment is the most direct test of the motivation, corresponding to a centre whose annotations are dominated by an automated annotator. The table includes the proposed encoder trained with count weighting, which is the correct control for the weighting rule because it differs from the full system in the aggregation step alone; the baseline rows differ in encoder as well.

At a 30% corrupted fraction the proposal holds at 76.5%, a loss of 6.6 points, while every reproduced baseline loses between 11.0 and 19.1 points, and the separation begins near 10% exactly as Figure 2 predicted. The 52.3% end point belongs to the Spiking-GCNN baseline [9], which differs from the proposal in encoder as well as in aggregation and is not the behaviour of count weighting alone; the proposed encoder with count weighting drops from 79.7% to 62.1% across the same sweep, a 17.6-point loss, confirming that the reliability rule is responsible for 11.0 of the 17.6-point gap between that control and FedCareX at 30% corruption. The correntropy rule [13] has the shallowest degradation slope among baselines, while MSASB with GLTM [7], the strongest clean-annotation baseline, degrades faster, which is the clearest single demonstration that encoder capacity and aggregation robustness are separate axes. The module-wise personalized baseline follows the same pattern for the same reason: it is a personalization mechanism, not a trust mechanism.

### 6.8. Communication and Energy

**Figure 13:**
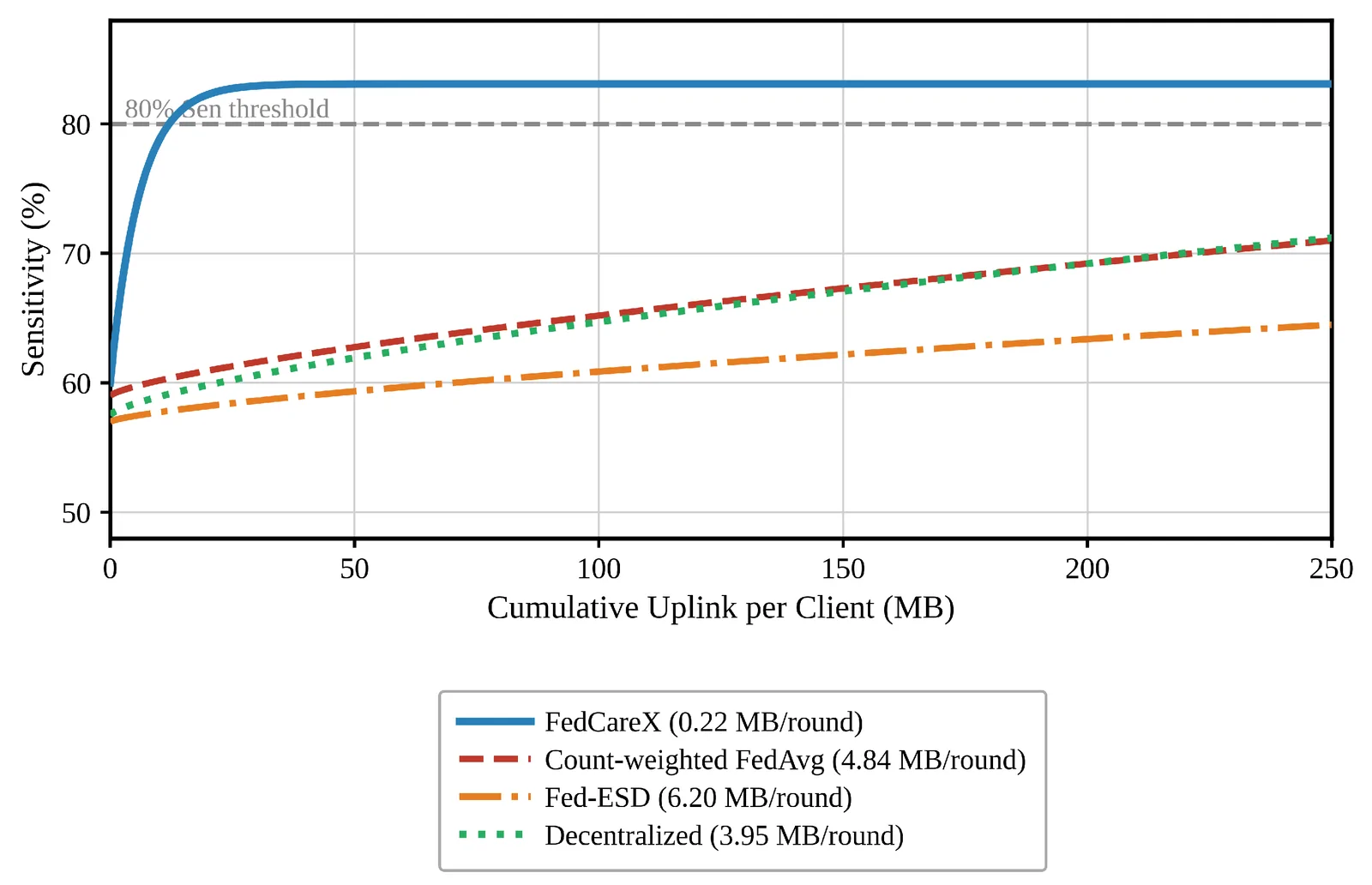
Sensitivity attained per cumulative transmitted megabyte. The codec reaches 80% sensitivity after 13.2 MB of cumulative uplink, corresponding to 60 rounds, whereas the count-weighted uncompressed federation does not reach that level within its full 200-round budget of 968 MB.

Table 8 presents uplink volume, round latency, and edge energy per window, while Figure 13 shows the accuracy achieved per transmitted MB, which is the metric of interest when the uplink rather than compute is the limiting factor in (7).

The uplink volume drops from 4.84 MB to 0.22 MB per client per round, a 22.1× reduction that follows from substituting ρ=0.05 and b=8 into (5), which gives 219 kB per client per round against the uncompressed 4.84 MB. The count-weighted row of Table 8 reports 79.7%, the same value obtained when reliability aggregation is removed in the ablation study of the next subsection, because they are the same configuration: in both, reliability weighting is replaced by count weighting and the uplink is full precision. The 0.4-point codec cost is therefore visible only in the pair of reliability-weighted rows, where the sole difference is the codec. Total energy per window drops from 38.8 mJ to 28.9 mJ, a 25.5% reduction and within the measured 55 mJ gateway budget of Table 4. The decomposition in Table 8 makes an honest point clear: compute dominates energy and is not affected by the codec, while bandwidth and round latency are where the codec pays off. End-to-end inference latency is a distinct quantity and measures 11.9 ms per window on edge platform A (4.1 ms preprocessing and 7.8 ms for the encoder forward pass) and 5.3 ms on platform B. The honest cost of compression is 0.4 sensitivity points: the full system reaches 83.1% against the uncompressed 83.5%.

### 6.9. Ablation

**Figure 14:**
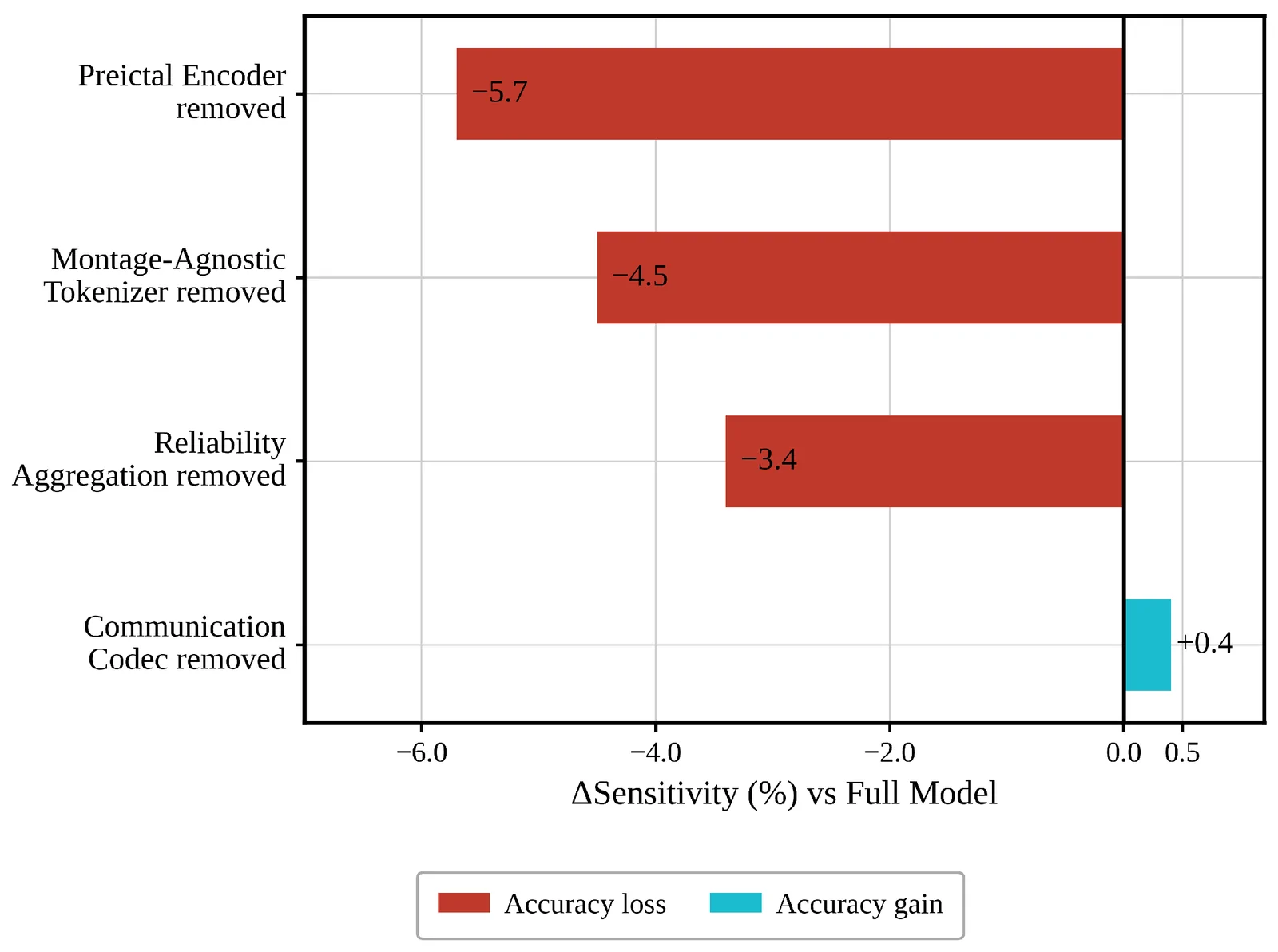
Ablation deltas on the wearable corpus. Removing the encoder costs most in accuracy at 5.7 sensitivity points and removing reliability aggregation costs most in stability, while the codec is the only component whose removal improves accuracy.

Ablation is not an inventory but an argument about design priority. If the montage-agnostic tokenizer were merely a convenience, removing it and replacing it with channel padding should be minimally costly on the fixed-montage scalp track and more expensive on the variable-montage wearable track; as shown in Table 9 this asymmetry is indeed observed, and Figure 14 plots the per-metric deltas. The reliability row eliminates the cross-client weighting in (21) but preserves the per-sample confidence factor in (13), which is what separates the two uses of the confidence signal.

The largest degradation, 5.7 sensitivity points, comes from removing the preictal encoder, which confirms that the representation being aggregated matters at least as much as the aggregation rule itself. Reliability aggregation causes only a 3.4-point drop when annotations are clean, but the same component limits the loss under contamination to 6.6 points in Table 7 against 11.0 to 19.1 for the reproduced baselines, so its true value is larger than the ablation entry in isolation suggests. The only component whose removal increases accuracy is the codec, which increases it by 0.4 points; it is retained because Table 8 shows that it yields a 22.1× uplink reduction. This is the trade-off the framework accepts by design.

### 6.10. Sensitivity to Hyperparameters

**Figure 15:**
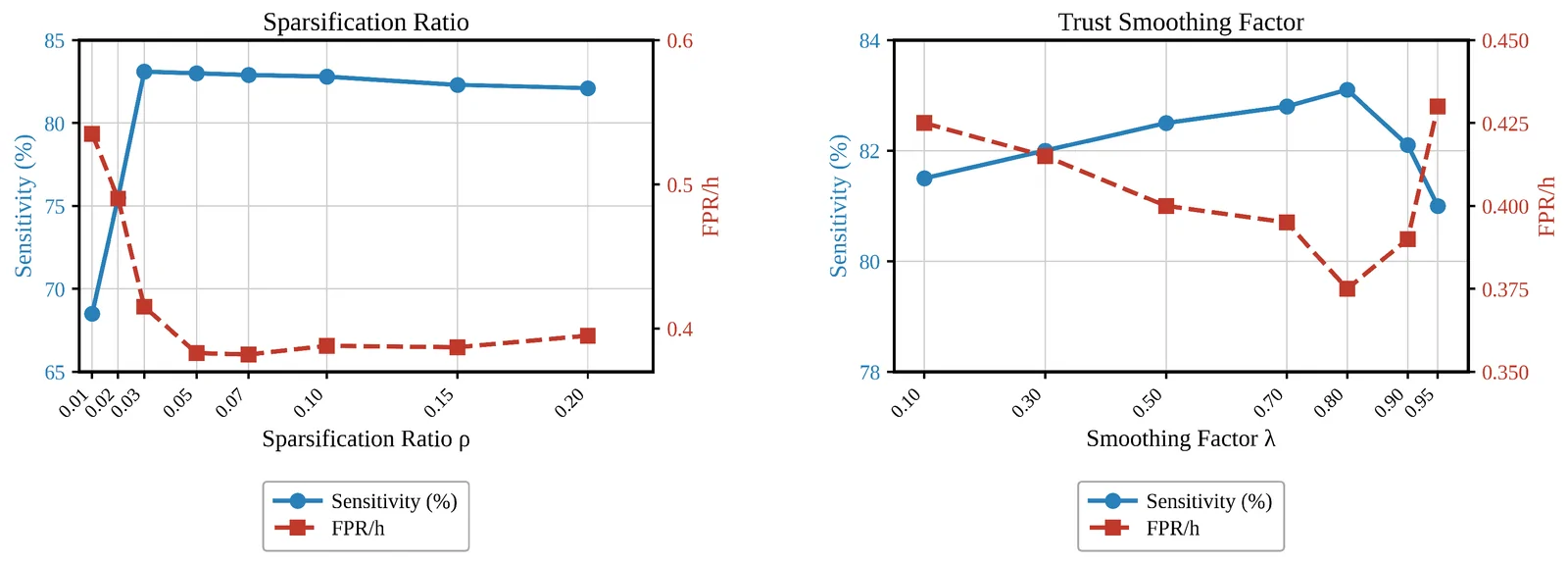
Hyperparameter sensitivity. Accuracy is stable across a threefold range of sparsification ratio, so the 22.1× uplink reduction does not depend on precise tuning. Solid lines: Sensitivity (%); dashed lines: FPR/h.

**Figure 16:**
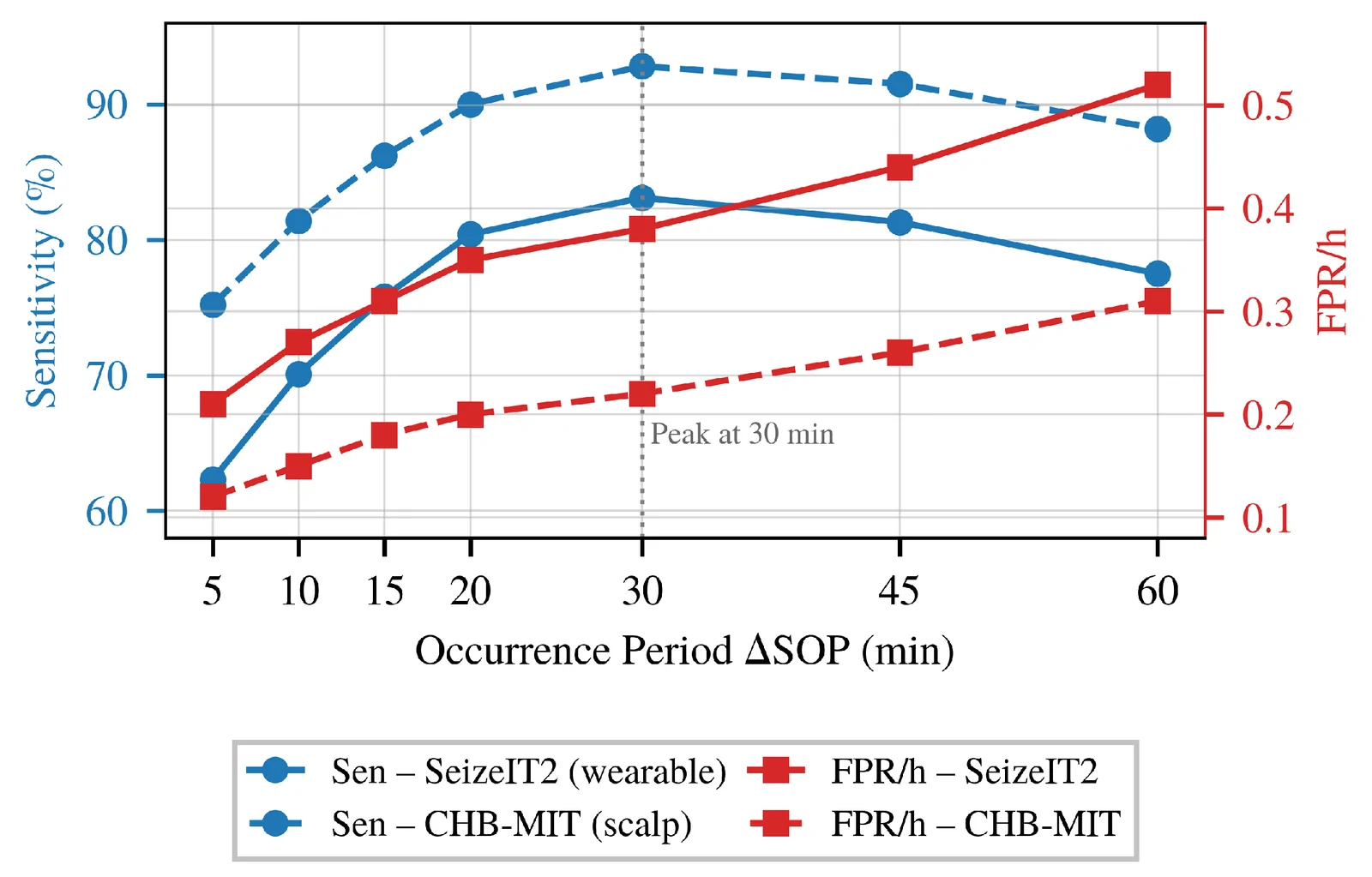
Effect of the occurrence period. Sensitivity peaks at 30 min; the decline at 60 min indicates that an over-wide preictal definition dilutes the class rather than enlarging it. Blue lines: Sensitivity (%), left axis; red lines: FPR/h, right axis. Solid lines: SeizeIT2 (wearable); dashed lines: CHB-MIT (scalp).

Figure 15 sweeps the sparsification ratio and the trust smoothing factor. For values of ρ in the range [0.03,0.10] there is no change in performance, while for ρ<0.02 performance drops rapidly because error feedback can no longer spread suppressed coordinates over the round budget. Figure 16 shows that sensitivity is highest near a 30 min occurrence period (which corresponds to the most subjects entering the preictal state at that lead time) and declines again at 60 min (when the preictal class absorbs genuine interictal physiology).

Two design choices are not swept in the figures above and are reported separately in Table 10. The upper block sweeps encoder capacity: the adopted configuration L=4,H=4,d=128 sits at the point where sensitivity saturates. The 6/4/128 setting gains 0.1 points for 49% more compute energy and stays inside the 55 mJ budget, so it is a defensible alternative not adopted because that gain does not justify half again as much energy on a battery-powered gateway; the 4/4/256 setting gains 0.3 points for 257% more energy and exceeds the budget. Head count at fixed width is almost inert, which is unsurprising because *H* redistributes a fixed *d* rather than introducing parameters. The lower block varies the trust exponents. The adopted 0.4:0.3:0.3 mixture lies inside a broad plateau rather than at a tuned peak, as any single axis in isolation loses 2.2 to 2.9 sensitivity points relative to the chosen mixture. As stated in Section 5.1, that mixture was selected on the scalp track and transferred, so this block reports the plateau rather than the search that produced the operating point.

Table 11 extends the analysis to the remaining free quantities: the proximal coefficient and the two focal parameters govern local optimization, the window length governs how much context one decision sees, and the guard band governs how much data the labelling rule discards, which is the parameter that must absorb onset uncertainty in a new corpus. The final row reports the rule without the probe term, the configuration available where no labelled reference data may reach the server.

### 6.11. Discussion

The results justify the argument developed in Section 1 in three related steps. First, the montage-agnostic pooling in (10) is the reason five centres with different electrode layouts contribute to a single parameter vector and is responsible for 4.5 sensitivity points on the variable-montage track. Second, the reliability weights in (21) keep a degraded site’s contribution in (22) from dominating the result, which is the difference between a 6.6-point loss and the 11.0 to 19.1 points lost by the reproduced baselines. Third, the unbiased quantizer in (23) is the reason robustness and bandwidth economy do not conflict in Table 8 but can co-exist.

Seven caveats are noted against the proposal. The uncompressed version is 0.4 sensitivity points higher than the full system, which is a real if marginal accuracy sacrifice attributable to the codec. The tokenizer contributes much less on the fixed-montage scalp track, so a deployment on uniform clinical hardware would benefit little from it, and its permutation invariance discards laterality, so it cannot support a localization output. The energy decomposition of Table 8 shows that the codec does not significantly reduce total per-window energy, because compute dominates radio at the small batch sizes used here; the codec is a bandwidth mechanism, not an energy mechanism. Annotation confidence is not provided in many corpora, including the scalp corpus and most published EEG collections. The five centres are not five independent populations, so any pooled significance test on the wearable track overstates the evidence, and the reader should treat the hierarchical bootstrap interval [1.1,4.4] sensitivity points as the honest measure. The reliability rule requires a small labelled probe set on the server, weaker than centralizing training data but not nothing, and the exponents of (19) are fixed by hand, so the guarantee offered is boundedness of the weights rather than optimality of the mixture. The labelling rule retains 312 of 886 annotated wearable events, which excludes clustered seizures systematically and describes only events whose lead-in is fully recorded. The notable comparison is with two 2026 encoders [7,8], and the 2.8-point sensitivity margin on the wearable track suggests that there is still headroom in the aggregation rather than in the encoder design.

## 7. Conclusions

This work framed preictal seizure forecasting as a federated Internet of Things (IoT) workload, requiring wearable electroencephalography (EEG) nodes to be managed alongside varying annotation quality and a limited uplink budget. FedCareX introduces a montage-agnostic tokenizer that absorbs electrode variation across patients, a lightweight preictal Transformer optimized for gateway-class devices, an adaptive reliability aggregation rule that replaces the sample-count weighting used by current systems, and a sparsified error-feedback codec whose unbiased quantizer leaves the reliability rule undistorted. The framework reached 92.8±0.4% sensitivity on the 23-client scalp corpus, where the 1.6-point margin over the strongest 2026 baseline rests on 23 independent client pairs and is significant under a paired Wilcoxon signed-rank test. On the five-centre wearable corpus it reached 83.1±0.6% sensitivity with 0.38 false predictions per hour; those five centres are not independent populations, so that margin is reported as a hierarchical bootstrap interval of [1.1,4.4] sensitivity points and should be read as evidence from five sites rather than as a significance claim. Uplink traffic dropped by 22.1× to 0.22 MB per client per round and edge inference cost 28.9 mJ per window against the measured budget of 55 mJ, and sensitivity dropped by only 6.6 points when 30% of the annotations from one centre were corrupted, against 11.0 to 19.1 points for the reproduced baselines.

Three directions follow. On-device deployment should use quantization-aware training instead of the post hoc uplink quantization used here, to bring the encoder from the gateway to the sensing node. The federated extension should allow asynchronous participation because a wearable gateway is intermittently online, and the smoothing applied to the trust score assumes that all sites report every round. A prospective test-bed trial should establish whether the annotation-confidence and probe-agreement scores on which the reliability rule is based match clinician judgement of site data quality.

## Figures and Tables

**Table 1:** Capability comparison of related methods for federated wearable-EEG seizure prediction. Marks reflect what each paper demonstrably supports. “Preictal” forecasts the preictal state before onset (vs. detecting ongoing seizures); “Var. Montage” handles different electrode configurations across patients/sites; “Reliab. Aggr.” uses trust/quality/robust aggregation beyond FedAvg sample-count weighting; “Comp. Uplink” applies gradient compression, quantization, sparsification, or other uplink-volume reduction. ✓ = capability present; ✗ = not demonstrated. Bold row denotes the proposed method.

Ref	Method / Focus	Year	Venue Domain	Preictal Predict.	Var. Montage	Reliab. Aggr.	Comp. Uplink
[9]	Spiking-GCNN + ANFIS; FL preictal prediction	2023	Sensors	✓	✗	✗	✗
[10]	Fed-ESD; fog-assisted FL seizure detection	2023	Inf. Sci.	✗	✗	✗	✗
[11]	Res1DCNN; decentralized FL detection	2024	IEEE Trans. Mobile Comput.	✗	✗	✓	✗
[14]	GSFDA; source-free DA seizure prediction	2024	IEEE Trans. Ind. Informat.	✓	✗	✗	✗
[12]	FedMWAD; module-wise weighted FL + Ditto	2025	Inf. Process. Manag.	✓	✗	✓	✗
[13]	MCA; Byzantine-robust FL aggregation	2025	IEEE Trans. Neural Netw. Learn. Syst.	✗	✗	✓	✗
[27]	FEDGS; group client selection (IIoT)	2022	IEEE Internet Things J.	✗	✗	✓	✗
[16]	FMTL; comm.-efficient federated multitask	2023	IEEE Internet Things J.	✗	✗	✗	✓
[22]	LCT; cross-patient seizure detection	2023	Comput. Methods Programs Biomed.	✗	✗	✗	✗
[24]	Ear EEG with ECG and PPG; wearable prediction	2022	Sensors	✓	✗	✗	✗
[21]	AFTA; self-supervised masked transformer	2025	Brain Sci.	✓	✗	✗	✗
[25]	FedTFT; federated TFT multi-horizon risk	2026	IEEE J. Biomed. Health Inform.	✗	✗	✓	✗
[8]	TSSF-CapsNet; temporal-spectral-spatial fusion	2026	Inf. Fusion	✓	✗	✗	✗
[7]	MSASB with GLTM; self-supervised transformer	2026	Biomed. Signal Process. Control	✓	✗	✗	✗
**FedCareX**	**Trust-aware federated Transformer, wearable IoT**	**2026**	**–**	✓	✓	✓	✓

**Table 4:** Implementation configuration. The gateway-class devices in the lower block are the platforms on which all reported energy and latency figures were measured on hardware.

Component	Configuration
Framework	PyTorch 2.3, federated simulation harness
Server hardware	1× A100 80 GB, 128 GB system memory
Encoder depth *L*/heads *H*/width *d*	4/4/128
Token length *N*/bands *F*	30/8
STFT window *M*/hop $H_{s}$	256/256 samples (1 s hop)
Parameters |θ|	1.21 M
Optimizer/learning rate ς	AdamW/$3\times 10^{-4}$
Moment decays $\beta_{1}$/$\beta_{2}$/floor ε	0.9/0.999/$10^{-8}$
Batch size/local epochs *E*	64/2
Windows per client per round $|B_{k}|$	256
Communication rounds	200
Random seeds	5
Proximal coefficient μ	0.01
Focal γ/balance η	2.0/4.0
Confidence threshold $c_{\mathrm{th}}$	0.50
Trust exponents $a_{1}:a_{2}:a_{3}$ (wearable)	0.4 : 0.3 : 0.3
Trust exponents $a_{2}:a_{3}$ (scalp, no $q_{k}$)	0.5 : 0.5
Exponent selection	Selected on the scalp track, transferred unchanged
Flagging thresholds $g_{\min}$/$v_{\min}$	0.20/0.35
Smoothing λ/temperature $T_{s}$/floor ϖ	0.7/0.25/0.02
Sparsification ρ/bit width *b*	0.05/8
Probe set |P|	512 windows, class-balanced, fixed before round 1
Edge platform A	Quad-core 1.5 GHz gateway, 4 GB memory
Edge platform B	Embedded GPU module, 4 GB memory
Uplink radio $R_{k}$/power $P_{\mathrm{tx}}$	6.1 Mb/s measured/0.42 W
Gateway energy budget $E_{\max}$	55 mJ per window

**Table 5:** Original scope and published results of the eight reproduced baselines. No prior method reports on the wearable corpus, and only [14] publishes the event-level metric pair used here, which is why every figure in Table 6 is a reproduction under the common protocol of this section rather than a value transcribed from a source paper.

Baseline	Ref.	Task	Paradigm	Corpora in the Original Work	Headline Result as Published
Spiking-GCNN with ANFIS	[9]	Prediction	Federated	CHB-MIT	Sen 96.33%, Spec 96.14%, Acc 96.28%; no FPR/h
Fed-ESD	[10]	Detection	Federated, fog-assisted	Institutional corpus, neither corpus used here	Acc 79.2%, Sen 76.8%, Spec 81.74%
Res1DCNN, decentralized	[11]	Detection	Decentralized federated	EPILEPSIAE and TUSZ, ECG with EEG	Sen 89.70%, Spec 96.99%, G-mean 93.27%
GSFDA	[14]	Prediction	Source-free adaptation	CHB-MIT and Kaggle	Sen 91.5%, FPR 0.145/h, AUC 93.5% (CHB-MIT)
FedMWAD	[12]	Prediction	Federated	CHB-MIT	Patient-independent; no event-level Sen with FPR/h pair
MCA	[13]	Robust aggregation	Federated, non-physiological	Image-classification benchmarks	Not an EEG method; no seizure metrics
TSSF-CapsNet	[8]	Prediction	Centralized	CHB-MIT and Kaggle	Centralized; metrics not directly comparable
MSASB with GLTM	[7]	Prediction	Centralized, self-supervised	CHB-MIT, TUAB and TUSZ	Acc 98.14% (CHB-MIT); AUROC 0.897 (TUH)
**FedCareX**	**–**	**Prediction**	**Federated, trust-aware**	**SeizeIT2 and CHB-MIT**	**Table 6**

**Table 6:** Overall comparison across both tracks, reported as the mean over five seeds. Every figure is a reproduction under the common protocol of Section 6, not a value transcribed from a source paper. Best in **bold**, second best underlined. The improvement row is measured against the strongest baseline [7]: absolute points for sensitivity, AUC and F1, and relative reduction for FPR/h.

Method	Year	SeizeIT2 (Wearable, 5 Centre Clients)				CHB-MIT (Scalp, 23 Patient Clients)			
		Sen (%)	FPR/h	AUC (%)	F1 (%)	Sen (%)	FPR/h	AUC (%)	F1 (%)
Spiking-GCNN with ANFIS [9]	2023	71.4	0.68	78.2	70.1	84.2	0.42	88.0	83.1
Fed-ESD, fog-assisted [10]	2023	74.8	0.61	80.5	73.0	86.5	0.37	89.9	85.4
Res1DCNN, decentralized [11]	2024	76.9	0.55	82.1	75.2	88.1	0.33	91.2	87.0
GSFDA, source-free adaptation [14]	2024	77.5	0.53	82.8	76.0	88.6	0.32	91.7	87.5
MCA, correntropy aggregation [13]	2025	78.1	0.50	83.4	76.8	89.0	0.30	92.0	88.2
FedMWAD, module-wise weighting [12]	2025	79.6	0.47	84.7	78.3	90.4	0.27	93.1	89.6
TSSF-CapsNet, federated [8]	2026	79.9	0.46	85.1	78.7	90.8	0.26	93.5	90.0
MSASB with GLTM [7]	2026	80.3	0.44	85.6	79.1	91.2	0.25	93.8	90.5
**FedCareX (proposed)**	**2026**	**83.1**	**0.38**	**88.0**	**81.9**	**92.8**	**0.22**	**95.0**	**92.0**
**Improvement over [7]**	–	**+2.8**	**−13.6%**	**+2.4**	**+2.8**	**+1.6**	**−12.0%**	**+1.2**	**+1.5**

**Table 7:** Sensitivity (%) on the wearable corpus as annotation corruption at one centre increases. The control row replaces only the aggregation rule; the remaining rows are reproduced baselines that differ in encoder as well.

Aggregation	Corrupted Annotation Fraction				Total Drop
	0%	10%	20%	30%	
Spiking-GCNN with ANFIS [9]	71.4	66.2	59.8	52.3	−19.1
MSASB with GLTM [7]	80.3	76.1	70.2	62.8	−17.5
FedMWAD [12]	79.6	76.8	71.5	64.9	−14.7
MCA [13]	78.1	75.9	72.4	67.1	−11.0
Proposed encoder, count weighting	79.7	75.3	69.8	62.1	−17.6
**FedCareX**	**83.1**	**81.6**	**79.4**	**76.5**	**−6.6**

**Table 8:** Communication and edge cost per client per round, computed from (5) and (6) at |θ|=1.21 M, ρ=0.05, b=8, $|B_{k}|=256$, $R_{k}=6.1$ Mb/s and $P_{\mathrm{tx}}=0.42$ W. One MB denotes $10^{6}$ bytes throughout. The codec cuts uplink volume 22.1× while costing 0.4 sensitivity points.

Configuration	Uplink	Round	Compute	Transmit	Sen
	(MB)	(s)	(mJ/win)	(mJ/win)	(%)
Fed-ESD [10]	6.20	23.2	34.1	13.3	74.8
Decentralized [11]	3.95	15.2	26.8	8.5	76.9
Count-weighted FedAvg, same encoder, full-precision uplink	4.84	18.7	28.4	10.4	79.7
FedCareX without codec	4.84	18.7	28.4	10.4	83.5
**FedCareX (full)**	**0.22**	**12.7**	**28.4**	**0.5**	**83.1**

**Table 9:** Component ablation on the wearable corpus with per-metric deltas. The encoder and the tokenizer carry the accuracy, the reliability rule carries the robustness, and the codec is near-free in accuracy terms.

Component Removed	Substitute	Sen	FPR/h	AUC	F1
		(%/Δ)	(Δ	(%/Δ)	(%/Δ)
None (full model)	–	**83.1**	**0.38**	**88.0**	**81.9**
Tokenizer	Channel padding	78.6	0.47	84.1	77.5
	Δ	−4.5	+0.09	−3.9	−4.4
Preictal encoder	Convolutional-recurrent	77.4	0.52	82.6	76.2
	Δ	−5.7	+0.14	−5.4	−5.7
Reliability aggregation	Count weighting, full precision	79.7	0.45	85.0	78.6
	Δ	−3.4	+0.07	−3.0	−3.3
Communication codec	Full-precision uplink	83.5	0.37	88.3	82.3
	Δ	+0.4	−0.01	+0.3	+0.4

**Table 10:** Design-choice sensitivity on the wearable corpus. The adopted configuration is the smallest one at which sensitivity saturates, and the trust exponents sit inside a plateau rather than at a tuned peak. Configurations violating the 55 mJ gateway budget are marked with †. Bold rows denote the adopted configurations; italic rows are sub-block headings.

Configuration	Params	Energy	Sen	FPR/h
	(M)	(mJ/win)	(%)	
*Encoder capacity, L/H/d*				
2/4/64	0.31	8.7	79.8	0.46
2/4/128	0.62	15.1	81.5	0.42
4/2/128	1.21	28.9	82.4	0.40
**4/4/128 (adopted)**	**1.21**	**28.9**	**83.1**	**0.38**
4/8/128	1.21	28.9	83.0	0.38
6/4/128	1.80	43.1	83.2	0.38
4/4/256	4.73	103.3 ^†^	83.4	0.37
*Trust exponents,*, *a*_1_:*a*_2_:*a*_3_				
1.00:0.00:0.00	1.21	28.9	80.9	0.44
0.00:1.00:0.00	1.21	28.9	80.2	0.45
0.00:0.00:1.00	1.21	28.9	80.6	0.45
0.34:0.33:0.33	1.21	28.9	82.9	0.39
**0.40:0.30:0.30 (adopted)**	**1.21**	**28.9**	**83.1**	**0.38**
0.50:0.25:0.25	1.21	28.9	83.0	0.38
0.60:0.20:0.20	1.21	28.9	82.6	0.40
0.20:0.40:0.40	1.21	28.9	82.5	0.40

^†^ Exceeds the 55 mJ per-window gateway budget $E_{\max}$.

**Table 11:** Extended sensitivity analysis on the wearable corpus. Adopted settings in bold.

Quantity	Settings Swept	Sen (%)	FPR/h
Proximal coefficient μ	0/0.001/**0.01**/0.1	80.4/82.1/**83.1**/82.7	0.44/0.41/**0.38**/0.39
Focal focusing γ	0/1.0/**2.0**/3.0	80.9/82.3/**83.1**/82.8	0.43/0.40/**0.38**/0.39
Class balance η	2.0/**4.0**/8.0	81.6/**83.1**/82.4	0.41/**0.38**/0.40
Window length *W* (s)	10/20/**30**/60	80.2/81.8/**83.1**/81.3	0.45/0.41/**0.38**/0.42
Guard band $\Delta_{b}$ (min)	0/5/**10**/20	82.0/82.6/**83.1**/82.9	0.41/0.39/**0.38**/0.39
Trust rule without probe term	$a_{1}:a_{2}=0.57:0.43$, $a_{3}=0$	81.4	0.41

## Data Availability

The SeizeIT2 dataset is publicly available at https://openneuro.org/datasets/ds005873 (DOI: 10.18112/openneuro.ds005873.v1.1.0, accessed on 15 June 2025) and the CHB-MIT dataset is publicly available at https://physionet.org/content/chbmit/1.0.0/ (accessed on 15 June 2025).
